# Biallelic *PI4KA* variants cause neurological, intestinal and immunological disease

**DOI:** 10.1093/brain/awab313

**Published:** 2021-08-20

**Authors:** Claire G Salter, Yiying Cai, Bernice Lo, Guy Helman, Henry Taylor, Amber McCartney, Joseph S Leslie, Andrea Accogli, Federico Zara, Monica Traverso, James Fasham, Joshua A Lees, Matteo P Ferla, Barry A Chioza, Olivia Wenger, Ethan Scott, Harold E Cross, Joanna Crawford, Ilka Warshawsky, Matthew Keisling, Dimitris Agamanolis, Catherine Ward Melver, Helen Cox, Mamoun Elawad, Tamas Marton, Matthew N Wakeling, Dirk Holzinger, Stephan Tippelt, Martin Munteanu, Deyana Valcheva, Christin Deal, Sara Van Meerbeke, Catherine Walsh Vockley, Manish J Butte, Utkucan Acar, Marjo S van der Knaap, G Christoph Korenke, Urania Kotzaeridou, Tamas Balla, Cas Simons, Holm H Uhlig, Andrew H Crosby, Pietro De Camilli, Nicole I Wolf, Emma L Baple

**Affiliations:** 1 RILD Wellcome Wolfson Centre, University of Exeter Medical School, Exeter, UK; 2 Wessex Clinical Genetics Service, Princess Anne Hospital, Southampton, UK; 3 Department of Neuroscience, Yale University School of Medicine, New Haven, CT, USA; 4 Department of Cell Biology, Yale University School of Medicine, New Haven, CT, USA; 5 Program in Cellular Neuroscience Neurodegeneration and Repair, Yale University School of Medicine, New Haven, CT, USA; 6 Howard Hughes Medical Institute, Yale University School of Medicine, New Haven, CT, USA; 7 Research Branch, Sidra Medicine, Doha, Qatar; 8 College of Health and Life Sciences, Hamad Bin Khalifa University, Doha, Qatar; 9 Murdoch Children's Research Institute, The Royal Children’s Hospital, Parkville, Melbourne, Australia; 10 Institute for Molecular Bioscience, The University of Queensland, Brisbane, Australia; 11 Department of Surgery and Cancer, Imperial College London, London, UK; 12 IRCCS Istituto Giannina Gaslini, 16147 Genova, Italy; 13 Peninsula Clinical Genetics Service, Royal Devon and Exeter Hospital, Exeter, UK; 14 Wellcome Centre Human Genetics, University of Oxford, Oxford, UK; 15 New Leaf Center, Mt. Eaton, OH, USA; 16 Department of Ophthalmology, University of Arizona College of Medicine, Tucson, AZ, USA; 17 Akron Children’s Hospital, Akron, OH, USA; 18 West Midlands Clinical Genetics Service, Birmingham Women's and Children's Hospital, Birmingham, UK; 19 Department of Gastroenterology, Sidra Medicine, Doha, Qatar; 20 West Midlands Perinatal Pathology, Birmingham Women's and Children's Hospital, Edgbaston, Birmingham, UK; 21 Department of Pediatric Haematology-Oncology, Pediatrics III, University of Duisburg-Essen, Essen, Germany; 22 Institute for Human Genetics, University Hospital Essen, University Duisburg-Essen, Essen, Germany; 23 Department of Pediatrics, Sana Kliniken Duisburg, Germany; 24 Division of Pediatric Allergy and Immunology, Children’s Hospital of Pittsburgh, UPMC, Pittsburgh, USA; 25 Division of Genetic and Genomic Medicine, Children’s Hospital of Pittsburgh, UPMC, Pittsburgh, USA; 26 Department of Paediatrics, Division of Immunology, Allergy, and Rheumatology, UCLA, Los Angeles, CA, USA; 27 Amsterdam Leukodystrophy Center, Department of Child Neurology, Emma Children's Hospital, Amsterdam University Medical Center, VU University Amsterdam and Amsterdam Neuroscience, 1081 HV Amsterdam, The Netherlands; 28 Department of Functional Genomics, Centre for Neurogenomics and Cognitive Research, Vrije Universiteit Amsterdam, 1081 HV Amsterdam, The Netherlands; 29 Department of Neuropediatrics, University Children's Hospital, Klinikum Oldenburg, 26133 Oldenburg, Germany; 30 Department of Child Neurology and Metabolic Medicine, Center for Pediatric and Adolescent Medicine, University Hospital Heidelberg, D-69120 Heidelberg, Germany; 31 Section on Molecular Signal Transduction, Eunice Kennedy Shriver National Institute of Child Health and Human Development, National Institutes of Health, Bethesda, MD, USA; 32 Translational Gastroenterology Unit, NIHR Oxford Biomedical Research Centre, John Radcliffe Hospital, University of Oxford, Oxfordshire, UK; 33 Department of Paediatrics, University of Oxford, Oxfordshire, UK; 34 Oxford NIHR Biomedical Research Centre, Oxford, UK; 35 Kavli Institute for Neuroscience, Yale University School of Medicine, New Haven, CT, USA

**Keywords:** hypomyelinating leukodystrophy, multiple intestinal atresia, *PI4KA*, FAM126A, TTC7A

## Abstract

Phosphatidylinositol 4-kinase IIIα (PI4KIIIα/*PI4KA*/OMIM:600286) is a lipid kinase generating phosphatidylinositol 4-phosphate (PI4P), a membrane phospholipid with critical roles in the physiology of multiple cell types. PI4KIIIα’s role in PI4P generation requires its assembly into a heterotetrameric complex with EFR3, TTC7 and FAM126. Sequence alterations in two of these molecular partners, TTC7 (encoded by *TTC7A* or *TCC7B*) and FAM126, have been associated with a heterogeneous group of either neurological (FAM126A) or intestinal and immunological (TTC7A) conditions.

Here we show that biallelic *PI4KA* sequence alterations in humans are associated with neurological disease, in particular hypomyelinating leukodystrophy. In addition, affected individuals may present with inflammatory bowel disease, multiple intestinal atresia and combined immunodeficiency. Our cellular, biochemical and structural modelling studies indicate that *PI4KA*-associated phenotypical outcomes probably stem from impairment of PI4KIIIα-TTC7-FAM126's organ-specific functions, due to defective catalytic activity or altered intra-complex functional interactions.

Together, these data define *PI4KA* gene alteration as a cause of a variable phenotypical spectrum and provide fundamental new insight into the combinatorial biology of the PI4KIIIα-FAM126-TTC7-EFR3 molecular complex.

## Teaser

Salter *et al.* show that biallelic variants in *PI4KA—*which encodes the enzymatic core of the PI4KIIIα-TTC7-FAM126 complex—cause a clinically diverse disorder comprising neurological, intestinal and immunological abnormalities.

## Introduction

*PI4KA* encodes phosphatidylinositol 4-kinase alpha (PI4KIIIα), the mammalian phosphatidylinositol (PI) 4-kinase^1^ with the predominant role in the generation of phosphatidylinositol-4-phosphate (PI4P) at the plasma membrane.^2^^,^^3^ To maintain protein stability and enhance enzymatic function, PI4KIIIα forms a ternary complex with two proteins, tetratricopeptide repeat domain 7 A/B (TTC7A/B) and hyccin/protein FAM126B (FAM126A/B), which further homodimerizes for plasma membrane recruitment via protein EFR3 homolog A/B (EFR3A/B).^3-6^ PI4P and its metabolites {phosphatidylinositol-4,5-bisphosphate [PI(4,5)P_2_]/phosphatidylinositol-3,4,5-trisphosphate [PI(3,4,5)P_3_]} that are generated by this highly conserved complex undertake fundamental signalling roles in the plasma membrane and other organelles.^1^ PI4KIIIα functional impairment, due to either defective catalytic activity or complex assembly, may therefore be expected to have widespread impact on multiple tissues. Accordingly, germline *Pi4ka* loss of function variants that abolish PI4KIIIα activity are incompatible with embryonic development.^3^^,^^7^

PI4KIIIα has been shown to be particularly important during intestinal and brain development, including myelination.^7–9^ In adult mice, PI4KIIIα global conditional inactivation or acute pharmacological inhibition leads to severe intestinal inflammation and sudden death,^3^^,^^10^^,^^11^ while in humans, a single study identified biallelic *PI4KA* variants as a candidate cause of a severe autosomal recessive neurodevelopmental disorder. This disorder comprises perisylvian polymicrogyria, cerebellar hypoplasia and arthrogryposis, and was identified in a small nuclear family with three female foetuses from pregnancies terminated before 34 weeks gestation.^12^ Interestingly, similar organ-specific disease outcomes have been identified in patients with biallelic pathogenic variants in the genes encoding the other PI4KIIIa complex proteins. This includes sequence alterations in *FAM126A* associated with a hypomyelinating leukodystrophy, termed hypomyelination and congenital cataracts,^13–17^ and loss of function alterations in *TTC7A* associated with a spectrum of bowel and immunological diseases ranging from early-onset inflammatory bowel disease (IBD) to multiple intestinal atresia (MIA), with or without combined immunodeficiency.^18–20^ So far, *TTC7A* variants remain the only known genetic cause of MIA in humans. Here we describe a clinically variable spectrum of neurological, immunological and intestinal (including MIA) disease associated with biallelic *Pi4ka* gene alterations.

## Materials and methods

### Clinical studies

**Figure 1:**
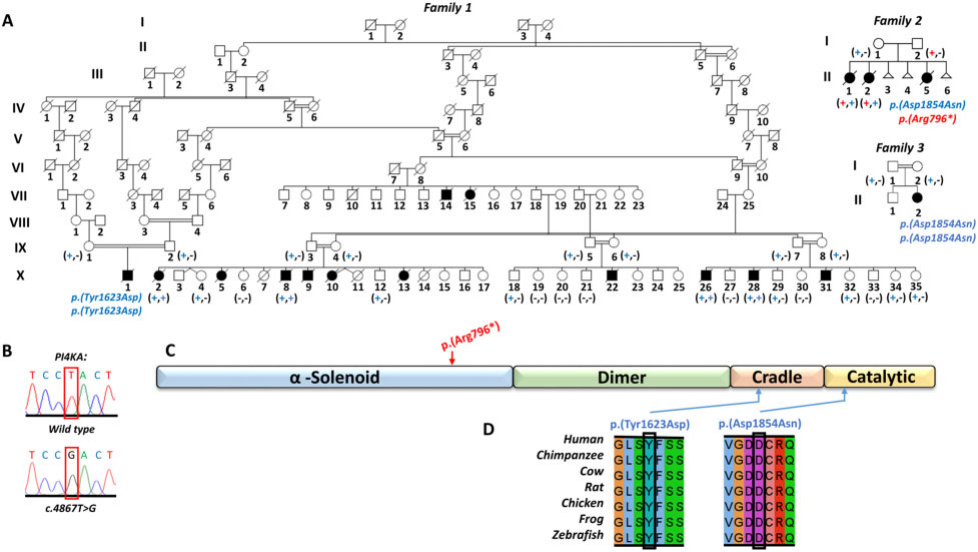
**Family pedigrees in which either the PI4KIIIα p.(Tyr1623Asp) or p.(Asp1854Asn) substitutions are associated with disease.** (**A**) Simplified pedigrees of the extended Amish family investigated, showing segregation of the *PI4KA* c.4867T>G variant [a dash (–) represents the wild-type allele, a plus symbol (+) represents the c.4867T>G, p.(Tyr1623Asp) sequence alteration], the previously reported family with biallelic *PI4KA* variants,^12^ and an additional family with the same p.(Asp1854Asn) missense variant in *PI4KA*. Shading indicates affected individuals. (**B**) Electropherograms showing the DNA sequence at the position of *PI4KA* c.4867T>G in a wild type control and a homozygous affected individual. (**C**) Schematic showing location of *PI4KA* variants (red = putative loss of function, blue = missense) with regard to PI4KIIIα functional domain (α-solenoid, dimer, cradle and catalytic) architecture. (**D**) Multiple species sequence alignment encompassing the amino acid residues affected by each missense alteration.

Research was performed with informed consent from the study participants or their legal guardians, according to institutional and international guidelines for studies with human participants and materials. Institutional study approvals include: Akron Children’s Hospital (IRB 986876-3), University of Arizona (IRB 10-0050-010), University of Exeter Medical School, Vrije Universiteit (VU), University Medical Center (IRB 2010/267), University of Pittsburgh (IRB 2001009) and Sidra Medicine (IRB 1601002512). Affected individuals and family members were investigated according to routine clinical standards for the diagnosis of neurological disease, MIA, IBD (Porto criteria) and immunological disorders. Individuals X:1, 2, 8, 26 and 28 from Family 1 (Fig. 1A) underwent surgery during their clinical management. Bowel histology from four of these patients and from foetus Family 2-II:5 was re-examined in light of the genetic findings.

### Genetic investigations

Genetic studies were performed as part of clinical and/or research investigations dependent on clinical presentation and family history. DNA was extracted from blood, buccal samples or paraffin-embedded bowel tissue. Whole-exome sequencing (WES, Family 1-IX:3–8 and X:28; Family 3-I:1, 2 and II:2; Family 8-I:1, 2 and II:1) or whole genome sequencing (WGS, Family 1-IX:1, 2 and X:1; Family 4-I:1, 2 and II:3; Families 5, 6 and 7-I:1, 2 and II:1) was performed on Illumina platforms. Full sequencing methodology for each family is described in the Supplementary material. Variants were filtered based on call quality, segregation with disease, impact on gene function and allele frequency in population databases. In Family 1, additional variant filtering was performed against our in-house Amish population specific database. *De novo*, homozygous or compound heterozygous variants present in exons or adjacent intronic regions were evaluated, which identified the biallelic *PI4KA* variant(s) in each family. In all cases, variants were assessed for clinical correlation with phenotype. WES and haplotype analysis in Family 1-IX:3–8 was used to exclude variants in *TTC7A*, with all coding and splice junctions covered at 10× (mean depth 116 in parental samples, 86 in proband; coverage at 20× was 98% in parental samples, 95% in proband). Remaining compound heterozygous/homozygous variants present genome-wide in Individual X:28 were filtered against WES data from Individuals IX:3–6, taking coverage into consideration.

Unique primers were used for amplification and bidirectional dideoxy sequencing of all *PI4KA* variants identified.

Genetic investigation methodology for Family 2 can be found in the original publication by Pagnamenta *et al.*^12^

### Cellular and molecular studies

#### Protein expression and purification

Plasmid gene constructs encoding components of the human PI4KIIIα complex were as follows: pcDNA3.1–3×FLAG-PI4KIIIα (1–2102, wild-type or Tyr1623Asp), pCMV6-AN-His-TTC7A (1–858) or TTC7B (1–843) and EGFP-FAM126A (2–289). In some experiments, pCMV-AN-His-GFP-EFR3B (mouse 9–817) was also expressed. Cotransfection of these constructs into Expi293F cells and induction of protein expression was performed following the vendor’s instructions (GIBCO). Then, 72 h after induction, cells were harvested and solubilized by resuspension in lysis buffer [50 mM Tris HCl, pH 8.0, 200 mM NaCl, 10% glycerol, 0.5 mM TCEP, 1× Complete EDTA-free protease inhibitor (Roche) and 0.5% Triton X-100].^6^ The suspension was incubated (ice, 10 min) and insoluble debris pelleted (17 000*g* for 20 min). The supernatant was incubated with anti-FLAG M2 beads (Sigma) at 4°C (2 h). Then, beads were washed with 10 bed volumes of wash buffer (lysis buffer lacking Triton X-100), incubated in two bed volumes of wash buffer with 1 mM ATP and 1 mM MgCl_2_ for 16 h at 4°C to remove chaperones and washed in 10 bed volumes of wash buffer, before elution in wash buffer containing 0.1 mg/ml 3×FLAG tag peptide (Sigma).

To detect the levels of protein expression, Expi293 cell lysates were analysed by western blotting (antibodies; anti-FLAG M2 antibody and anti-His antibody; Sigma, and anti-GFP antibody; Abcam), using ImageJ. Quantification of the western blot was performed by comparing lysates from cells expressing (i) wild-type PI4KIIIα, TTC7A and FAM126A; and (ii) mutant PI4KIIIα, wild-type TTC7A and wild-type FAM126A. Results were expressed as percentages, with expression levels of the complex subunits from the cells expressing all wildtype proteins set at 100% and the corresponding subunit expression levels in cells containing the mutant PI4KIIIα molecule calculated as a percentage of this. This methodology was then repeated in cells expressing TTC7B in place of TTC7A.

Analysis of endogenous levels of TTC7A and TTC7B in total homogenates of human brain and intestine (from Zyagen) was performed by western blotting (anti-TTC7A; Origene, anti-TTC7B; Abcam).

#### Lipid kinase assay

The concentration of purified PI4KIIIα complex was determined by OD reading at 280 nm, with enzymatic activity assessed with the ADP-Glo lipids kinase system (Promega) using 0.1 mg/ml lipid substrate and 25 μM ATP. The reaction was started by the addition of 5 nM PI4KIIIα complex and carried out at room temperature for 10 min.

#### Electron microscopy

Copper grids overlaid with 10 nm amorphous carbon were glow discharged at 25 mA in residual air for 30 s. Purified PI4KIIIα complex was diluted with elution buffer to match the concentration of the least concentrated sample (20 nM). Next, 4 µl of each protein sample was applied twice to a glow-discharged grid, incubated for 30 s after each application, then stained with 2% uranyl acetate, blotted and dried. Micrographs were collected on an FEI Tecnai T12 electron microscope with an operating voltage of 120 kV at ×52 000 magnification (pixel size = 2.1 Å).

### Molecular modelling studies

Molecular modelling of missense variants identified in all eight families was performed to explore the potential impact of each mutation on complex formation with TTC7A/B and PI4KIIIα enzymatic function. The full methodology is described in the Supplementary material.

### Data availability

The authors confirm that the data supporting the findings of this study are available within the article and/or its Supplementary material. On reasonable request, genetic data can be made available.

## Results

### PI4KIIIα p.(Tyr1623Asp) substitution is associated with autosomal recessive MIA and immunodeficiency

**Figure 2:**
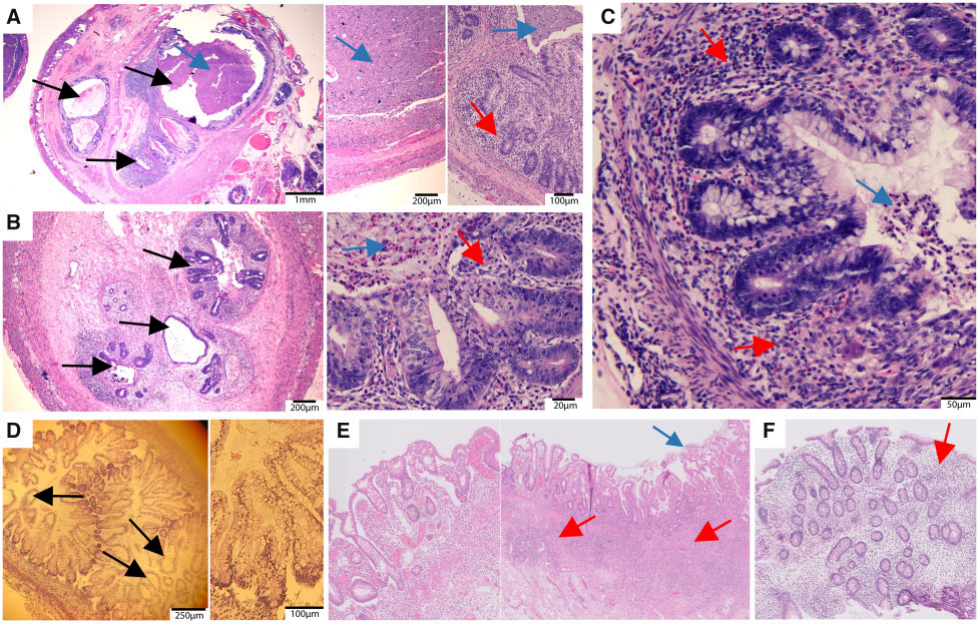
**Bowel histology from affected individuals with biallelic *PI4KA* variants.** (**A**–**C**) Bowel sections from Amish infants with MIA, showing multiple distinct small lumens, each of which is lined by mucosa and surrounding muscularis mucosa. There is variable dilated and narrowed lumen containing partially calcified meconium, with areas of mucosal atrophy and foci of acute neutrophilic mucosal inflammation. There are focal collections of mucosal and submucosal histiocytes with associated calcified material. The ganglion cell populations are normal. (**D**) Autopsy tissue from a foetus (Family 2-II:5) at gestational Week 16 showing ballooned epithelial cysts. (**E**) Large bowel section from affected proband (Family 5-II:1) showing colitis with transmural lymphocytic inflammation, epithelial damage and luminal cellular detritus. (**F**) Large bowel section from affected proband (Family 4-II:3) showing lymphocytic active inflammation of the colon. Images represent haematoxylin and eosin-stained tissue sections obtained at routine endoscopies or autopsy. Black arrows show cystic luminal structures, red arrows show inflammatory infiltrate, blue arrows indicate excessive luminal cell detritus and/or crypt abscesses.

We initially investigated the cause of disease in 12 infants from four nuclear Amish families affected by a severe extensive MIA (Family 1-VII:14, 15, X:2, 5, 8–10, 13, 22, 26, 28, 31; Fig. 1A), presenting after birth with clinical signs of bowel obstruction including abdominal distension, bilious vomiting and failure to pass meconium. Although no antenatal scanning was performed, a clinical history compatible with third trimester polyhydramnios was obtained retrospectively. Explorative abdominal surgery in seven infants revealed multiple sections of abnormal, inflamed and atretic bowel from pylorus to anus. Detailed surgical records were available for four infants, all of whom had atresia affecting the small intestine and colon. Due to their historical nature, and cultural and financial constraints (Amish families would not typically accept bowel transplantation or other similar invasive medical procedures), these families primarily chose palliative care for affected children. One infant (Individual X:28), considered to have sufficient patent bowel for viable gut function, underwent multiple surgeries. This individual had 120 cm of patent but dilated proximal ileum, malrotation and volvulus, but with no acute ischaemic pathology. The colon showed multiple atresias with the longest patent section measuring 7.5 cm. In the remaining six children, an intra-operative decision for palliative care was taken due to the extent of atresia (see Fig. 2 and Supplementary Tables 1 and 2 for further clinical, surgical and histological findings). Subsequent affected siblings presenting with bowel obstruction after birth received home palliative care. Bowel histology (Individuals X:2, 8, 26 and 28) showed a consistent pattern of intestinal abnormalities, with multiple lumen anlagen and intestinal cysts, loss of epithelial layer, dense luminal cell detritus and a mild inflammatory infiltrate. There were numerous atretic segments with multiple distinct small lumens lined by mucosa and muscularis mucosa (sieve-like appearance), and sections with complete fibrous lumen obliteration (cord-like appearance) (Fig. 2A–C). Growth and head circumference measurements were normal, and no additional systemic or neurological abnormalities were identified. Affected infants did not tolerate enteral feeds and died within one month. While in-depth immunological investigations were not initiated and the short lifespans limited detection of clinical infection susceptibility, the small number of full blood count indices available were unremarkable, aside from neonate Individual X:28 with mild intermittent lymphopaenia and one episode of marked lymphopaenia.

To define the genetic cause of disease, genome-wide single nucleotide polymorphism mapping and WES were undertaken assuming homozygosity for a founder variant, although also considering other inheritance mechanisms. Because only paraffin-embedded tissue was available from affected infants and low-quality DNA yields, WES was performed on a single affected individual (Family 1-X:28) and six healthy parents (Individuals IX:3–8). This, as well as parental haplotype analysis across the *TTC7A* genomic region (data not shown), conclusively excluded *TTC7A* variants. Filtering of WES data using standard metrics (pass call quality, allele frequency <0.01 in gnomAD)^21^ identified a single potentially pathogenic missense alteration, which could not be excluded by cosegregation analysis, *PI4KA* [Chr22(GRCh37):g.21081592A>C, [rs776650691]; NM_058004.3: c.4867T>G; NP_477352.3: p.(Tyr1623Asp)]. This was confirmed by dideoxy sequencing and found to cosegregate as expected for an autosomal recessive disease in the four affected neonates from whom DNA was available (Individuals X:2, 8, 26, 28), 14 unaffected siblings and all living parents (Fig. 1A and B). The variant is predicted to substitute a highly conserved tyrosine residue in the armadillo repeat/cradle protein domain (Fig. 1C and D) and predicted deleterious by *in silico* tools (Supplementary Table 3). It is rare and present only in heterozygous fashion in gnomAD v.2.1.1 (allele frequency 0.000008). As expected for a founder variant, it is also present in our collaborative Anabaptist genetic disease variant database (Anabaptist Variant Server containing >10 000 individuals; see the 'Acknowledgements' section) at low allele frequency (0.0006) and only in heterozygous state.

The same homozygous c.4867T>G; p.(Tyr1623Asp) *PI4KA* alteration was subsequently detected by trio WGS in an infant of Amish ancestry who died at 3 weeks from MIA and severe immunodeficiency (Family 1-X:1; Fig. 1A). No putative pathogenic variants in *TTC7A* or other genes associated with primary immunodeficiency were identified. We were able to link this family to the other four Amish families using the Swiss Anabaptist Genealogical Association database.^22^ The child presented with recurrent emesis and failure to pass meconium, similar to the other Amish infants. Surgical exploration revealed malrotation of the small bowel and multiple areas of atresia affecting the small intestine and colon. Notably, immunological studies identified a severe T-cell lymphopaenia, affecting CD8^+^ T cells more than CD4^+^ T cells, moderate B- and NK-cell lymphopaenia and agammaglobulinaemia. T-cell proliferation in response to mitogen stimulation was intact. No neurological anomalies were noted, and cranial ultrasound scan was unremarkable. As with the previous families, the parents opted for palliative care. Clinical, surgical and immunological findings, and WES/WGS variants are summarized in Supplementary Tables 1–3 and the Supplementary material.

### PI4KIIIα p.(Asp1854Asn) substitution is associated with CNS abnormalities, IBD and immunodeficiency

Previously only one family had been reported in whom biallelic *PI4KA* gene variants had been identified as a candidate cause of disease. Pagnamenta and colleagues described three affected foetuses with predominantly neurological disease (perisylvian polymicrogyria, cerebellar hypoplasia and arthrogryposis).^12^ As there is only minimal myelin present in defined structures in healthy foetuses, it was not possible to determine hypomyelination. All three foetuses were compound heterozygous for a *PI4KA* p.(Arg796*) nonsense alteration predicted to undergo nonsense mediated decay, and a missense p.(Asp1854Asn) variant in the catalytic domain (Family 2; Fig. 1A).^12^ In contrast to the Amish infants, no intestinal pathology was described. We therefore re-evaluated archived histological specimens from the affected foetuses, which revealed ballooned epithelial cysts, although no obstructions or multiple lumen were identified (Fig. 2D; intestinal samples from two foetuses showed autolysis, preventing assessment).

**Figure 3:**
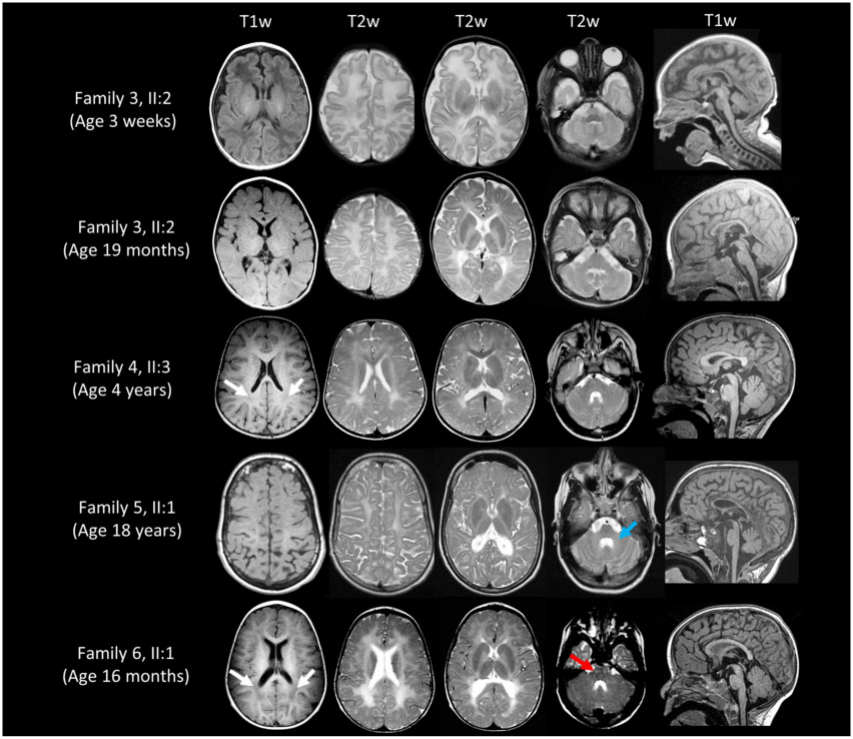
**Neuroimaging from affected individuals with biallelic *PI4KA* variants.** All affected individuals show a T_2_ hyperintense signal of the supratentorial white matter, very similar to the signal of unmyelinated white matter in a neonate (Family 3 at age 3 weeks). In Family 6-II:1, this signal is relatively high, with areas of relatively hypointense T_1_ signal (white arrows) and the pyramidal tracts show a T_2_ hyperintense signal in the brain stem (red arrow). The middle peduncles of the cerebellum and the cerebellar white matter may be affected (blue arrow), with a hyperintense T_2_ signal. The sagittal images show a thin corpus callosum in the oldest affected individual (Family 5-II:1) and in the patient from Family 3 (II:2), and only very mild cerebellar atrophy. The patient from Family 3, also has immature gyration at age 3 weeks, with myelination of the posterior limb of the internal capsule (PLIC; this is normal at that age). At age 19 months, the previously normal signal of the PLIC is now T_2_ hyperintense, as is the signal of the rest of the white matter. The T_2_ signal of the pallidum is also too high, indicating lack of normal myelination of this grey matter structure. There is no evidence of polymicrogyria in this patient, although gyration especially of the frontal lobes has not fully developed. The cerebellum and pons are slightly too small already in the neonatal period, and also at age 19 months (also compared with the patient from Family 6 at age 16 months).

Further insight into the clinical outcomes associated with the *PI4KA* p.(Asp1854Asn) missense alteration is provided by a Turkish child who was found to be homozygous for the same *PI4KA* variant (Family 3; Fig. 1A). This 13-year-old is severely microcephalic (−10 standard deviations) and presented from Day 1 of life with treatment-resistant seizures. She has severe global impairment, absent head control, central hypotonia and peripheral spasticity, bilateral optic atrophy, hearing loss and widespread small pigmented skin macules. Magnetic resonance neuroimaging at 3 weeks of age revealed immature gyration (without evident polymicrogyria) and a small cerebellum, and at 19 months immature gyration, hypomyelination, thin corpus callosum and mild cerebellar atrophy (Fig. 3). At age 2 years, she had status epilepticus due to hypoglycaemia, with subsequent atrophy of the right hemisphere and progressive cerebellar atrophy. While this child again displays a predominately neurological phenotype, notably at age 10 years she was found to be neutropenic and to have eosinophilia, B-cell lymphopaenia, hypogammaglobulinaemia and splenomegaly. She was subsequently diagnosed with a diffuse grade 3A follicular non-Hodgkin lymphoma (bcl6 translocation) more typically seen in adulthood, but which responded well to treatment with rituximab. Recent immunological investigations revealed a persistent moderate lymphopenia with marked reduction in B and NK cells and poor antibody response to previous immunization. There was no previous history of recurrent infections or overt gastrointestinal symptoms. However, she has a long-standing iron deficient anaemia that has not resolved despite supplementation. The child’s clinical condition has prevented a full gastrointestinal assessment, but a recent stool sample revealed an elevated calprotectin of 829 µg/g (<50), which, in combination with treatment-resistant anaemia, is highly suggestive of undiagnosed IBD (see Supplementary Table 1 and Supplementary material for detailed clinical information).

### The PI4KIIIα p.Tyr1623Asp alteration affects complex formation with TTC7A, providing a molecular rationale for tissue-specific phenotypic outcomes

The p.(Asp1854Asn) missense alteration defined by Pagnamenta *et al*.^12^ and in Family 3 presented here, affects a highly conserved residue within the PI4KIIIα catalytic domain. Our previously published functional evaluation of the p.(Asp1854Asn) alteration determines that although the mutant PI4KIIIα protein is stable, the Asp1854Asn substitution results in near undetectable levels of PI4KIIIα enzymatic activity *in vitro*.^12^

**Figure 4:**
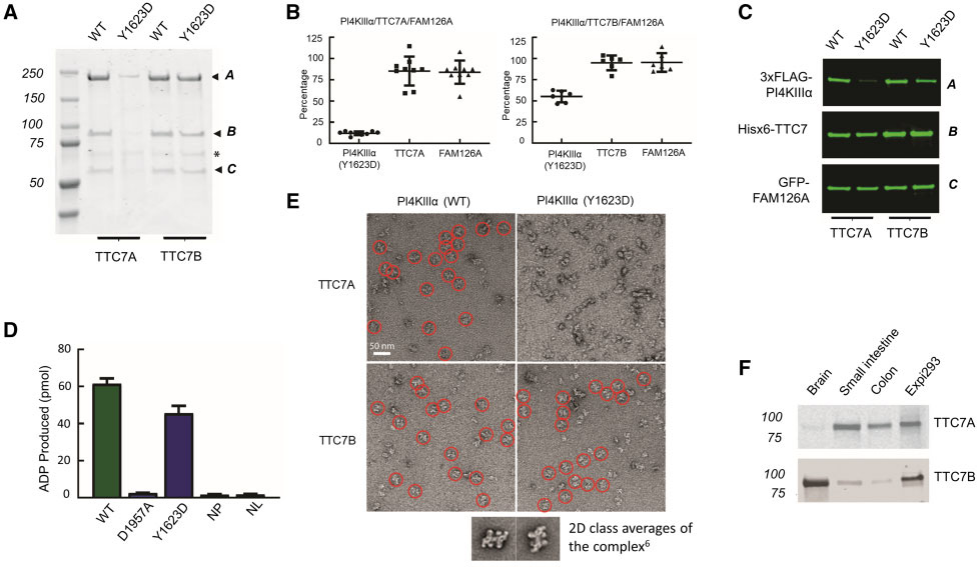
**Outcomes of PI4KIIIα p.Tyr1623Asp sequence variant on complex formation with TTC7A or TTC7B.** (**A**) PI4KIIIα complexes purified by anti-FLAG resin and analysed by SDS-PAGE and Coomassie Blue staining. Both TTC7A and TTC7B form complexes with wild-type (WT) PI4KIIIα and FAM126A. With mutant PI4KIIIα, abundant complex containing TTC7B is observed, but very little complex is detected in TTC7A expressing cells (arrowheads: A, 3xFLAG-PI4KIIIα; B, Hisx6-TTC7A/7B; C, GFP-FAM126A). (**B**) Reduced levels of mutant (Tyr1623Asp) PI4KIIIα when co-expressed in Expi293 cells with other complex subunits, assessed by quantification of western blots (shown in **C**). The percentage of each subunit relative to cells expressing wild-type complex components is shown. *Left*: TTC7A-containing complex (*n* = 9); *right*: TTC7B-containing complex (*n* = 6). Levels of mutant PI4KIIIα were reduced relative to wild-type when co-expressed with TTC7B or TTC7A, but much more strongly when co-expressed with TTC7A (∼11% of wild-type). (**C**) Levels of each complex subunit assessed by western blotting on co-expression of PI4KIIIα (wild-type or Tyr1623Asp), TTC7 (TTC7A or TTC7B) and FAM126A in Expi293 cells. Equal amounts of total protein were loaded on each lane (A; anti-FLAG, B; anti-His, C; anti-GFP). (**D**) Kinase activity assay of the PI4KIIIα-TTC7B-FAM126A complex, with activities measured by the ADP-Glo kinase assay (*n* = 3). The mutant complex displayed moderately reduced activity (∼70% of wild-type). Negative control: kinase with catalytic site mutation Asp1957 (NP; no protein, NL; no lipids). (**E**) Electron micrographs of negatively stained purified PI4KIIIα-TTC7-FAM126A complexes containing wild-type or mutant PI4KIIIα. Scale bar = 50 nm. *Insets* (shown below main image) display the appearance of previously described 2D class averages of the wild-type complex (reproduced from Lees *et al*.^6^). Particles reflecting this expected complex morphology are clearly visible in cells expressing wild-type or mutant PI4KIIIα and TTC7B (red circles), but are not visible in cells expressing the mutant PI4KIIIα and TTC7A. (**F**) Western blots showing expression of TTC7A and TTC7B in human brain, and small and large intestine. Equal amounts of tissue lysates were loaded onto each lane for both blots. Positive controls: Expi293 cell lysates overexpressing TTC7A or TTC7B. Uncropped gel images for **A**, **C** and **F** are available in the Supplementary material.

While the PI4KIIIα catalytic subunit is encoded by a single gene (*PI4KA*), the other two complex subunits enabling PI4KIIIα stability are each encoded by two genes generating FAM126A/B and TTC7A/B isoforms; each molecular isoform has largely overlapping cellular function, but different tissue distributions.^3^^,^^6^^,^^14^^,^^23^^,^^24^ We therefore hypothesized that the phenotypic spectrum of gastrointestinal, neurological and immunological disease associated with *PI4KA* p.(Tyr1623Asp)/p.(Asp1854Asn) variants may stem from impairment of particular molecular roles requiring organ-specific PI4KIIIα-TTC7-FAM126 complex functional interactions. Unlike the PI4KIIIα p.(Asp1854Asn) variant we studied previously,^12^ the PI4KIIIα Tyr1623 residue lies outside the catalytic site, but close to the TTC7 molecular interface. The striking phenotypical overlap between TTC7A deficiency patients and the MIA and immunodeficiency affecting the Amish neonates indicates that the p.(Tyr1623Asp) substitution may specifically impair PI4KIIIα-TTC7A binding. As no fresh tissue and only very limited amounts of paraffin-embedded tissue were available, protein expression studies in affected tissue were not possible. To explore the impact of the p.(Tyr1623Asp) alteration on complex formation and catalytic activity, we co-expressed plasmids encoding components of the human PI4KIIIα complex [pcDNA3.1–3×FLAG-PI4KIIIα (1–2102, wild-type or Tyr1623Asp), pCMV6-AN-His-TTC7A (1–858) or TTC7B (1–843), and EGFP-FAM126A (2–289)] in Expi293 cells, and purified the complex. In some experiments pCMV-AN-His-GFP-EFR3B (mouse 9–817) was also expressed. We initially confirmed that both TTC7A and TTC7B can assemble with PI4KIIIα and FAM126, as previously only the TTC7B complex had been purified (Fig. 4A),^6^^,^^14^^,^^24^ and that both complexes copurify with EFR3 (Supplementary Fig. 1) enabling membrane targeting.^3^^,^^4^ In addition, we showed that PI4KIIIα-TTC7A-FAM126A enzymatic activity is comparable with that of the PI4KIIIα-TTC7B-FAM126A complex (Supplementary Fig. 2). Next, we examined expression levels of TTC7, FAM126 and either wild-type or mutant p.Tyr1623Asp PI4KIIIα when co-expressed in Expi293 cells. Western blots of cell lysates revealed that levels of the mutant kinase were reduced by ∼90% relative to wild-type when co-expressed with TTC7A and by ∼50% when co-expressed with TTC7B, although levels of TTC7 (either A or B) and FAM126 were not substantially affected by the expression of mutant PI4KIIIα in place of the wild-type kinase (Fig. 4B and C). These results indicate the p.Tyr1623Asp alteration affects the stability of PI4KIIIα far more strongly when co-expressed with TTC7A, than when expressed with TTC7B. Further supportive evidence for this conclusion was provided by complex purification in transfected Expi293 cells. Robust amounts of protein complex were obtained from cells expressing either wild-type or mutant p.Tyr1623Asp PI4KIIIα in the presence of TTC7B and the activity of the mutant complex was only modestly reduced relative to wild-type complex (Fig. 4D). In contrast, barely detectable levels of the mutant complex could be obtained from TTC7A expressing cells (Fig. 4A), and indeed its activity could not be tested due to these low yields. Taken together, these results indicate that mutant p.Tyr1623Asp PI4KIIIα is inherently unstable unless in a complex with TTC7 and FAM126, and that the mutant PI4KIIIα-TTC7B-FAM126 complex forms more readily or is more stable than its TTC7A containing counterpart. Negative stain electron microscopy of the purified complex further supports this interpretation (Fig. 4E). Particles resembling those previously described composed of PI4KIIIα-TTC7B-FAM126A complexes^6^ were readily identified in preparations of purified complexes obtained from cells expressing wild-type kinase with either TTC7A or TTC7B, and from cells expressing mutant kinase with TTC7B. However, such complexes could not be identified in preparations obtained from cells expressing mutant kinase with TTC7A. Thus, even the very small amount of complex purified from these cells appears to be unstable.

Together, the results of these biochemical studies provide insights into some of the probably multifaceted functional outcomes associated with the p.(Tyr1623Asp) substitution including a selective negative impact on PI4KIIIα-TTC7A interaction, resulting in significantly reduced complex formation and subsequent instability of PI4KIIIα. Thus, tissues primarily expressing TTC7A would likely be profoundly affected by the PI4KIIIα p.(Tyr1623Asp) mutant molecule, potentially explaining the severe gastrointestinal MIA outcomes seen in affected Amish neonates. This is supported by protein expression databases^23^ and western blotting of total human tissue homogenates, which confirm that TTC7A is expressed in small and large intestine at higher levels than in brain, while conversely TTC7B is expressed at higher levels in brain than intestine (Fig. 4F)*.* In contrast, the stability and relatively conserved function of the mutant PI4KIIIα-TTC7B-FAM126 complex may provide an explanation as to why the Amish neonates were not affected by severe antenatal/neonatal onset neurological disease, as seen in the previously described foetuses^12^ and the child in Family 3 (Individual II:2; Fig. 1A).

### Biallelic candidate *PI4KA* variants identified in individuals with hypomyelinating leukodystrophy, sometimes in association with IBD

**Figure 5:**
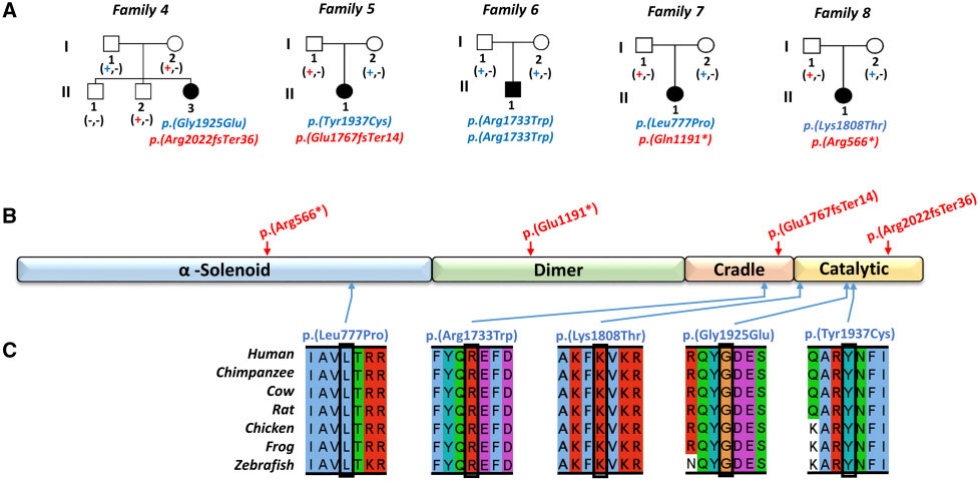
**Family pedigrees and biallelic *PI4KA* variants identified as a candidate cause of disease.** (**A**) Simplified pedigrees for the five affected individuals identified through our international collaboration, showing segregation of the biallelic *PI4KA* variants identified as a candidate cause of their clinical presentation [a dash (–) represents the wild-type allele, a plus symbol (+) represents the sequence alteration]. (**B**) Schematic showing location of *PI4KA* variants (red = putative loss of function, blue = missense) with regard to PI4KIIIα functional domain (α-solenoid, dimer, cradle and catalytic) architecture. (**C**) Multiple species sequence alignment encompassing the amino acid residues affected by each missense alteration.

We speculated that a broader spectrum of neurological, gastrointestinal and immunodeficiency phenotypes may stem from *PI4KA* gene variants dependent on the nature and location of each variant, and the extent to which complex binding, functional properties and catalytic activity of the encoded PI4KIIIα protein are affected. To investigate this and further characterize the genetic and clinical spectrum of *PI4KA*-related disease, we explored GeneMatcher and our international collaborative network. This identified five affected individuals from five nuclear families with rare, predicted damaging biallelic candidate *PI4KA* variants. These individuals (aged 5–24 years) presented with distinctive and overlapping clinical phenotypes (see Fig. 5A and Supplementary Tables 1–3 for detailed clinical reports, family pedigrees and WES/WGS variants). All five individuals presented with hypomyelinating leukodystrophy and two developed severe IBD. Neuroimaging, available in all individuals, identified diffuse elevation of T_2_-weighted signal in the supratentorial white matter, compatible with hypomyelination, as the most prominent and consistent feature. Three affected individuals (Families 4-II:3, 6-II:1 and 7-II:1) displayed areas of relatively T_1_-hypointense white matter signal, improving with age as seen in hypomyelination and congenital cataracts^25^ (Fig. 3 and Supplementary Fig. 3). Axial T_1_-weighted images from Family 8-II:1 were not available.

The proband from Family 4 (Individual II:3) presented at 6 weeks of age with severe colitis that spared the small intestine (Fig. 2F). At 2 years, she showed symptoms suggestive of a primary hypomyelination disorder, and by age 4 had spastic diplegia, global developmental delay and dystonic hand movements. Neuroimaging showed hypomyelination (Fig. 3 and Supplementary Fig. 3). Trio WGS revealed compound heterozygosity for frameshift (p.Arg2022GlnfsTer36 in exon 52 of 55, g.21065005delC, c.6065delG, gnomAD; absent) and missense (p.Gly1925Glu; g.21066802C>T, c.5774G>A, gnomAD; absent) variants, with the missense alteration predicted to impact the *PI4KA* catalytic domain (Fig. 5B). The affected individuals from Families 5–7 all presented with nystagmus, ataxia and lower limb spasticity, and magnetic resonance neuroimaging revealed hypomyelinating leukodystrophy reminiscent of Pelizaeus–Merzbacher-like disease (Fig. 3). One of these individuals (Family 5-II:1), developed severe ulcerative colitis (age 19 years) requiring total colectomy due to treatment-refractory disease (Fig. 2E). The other two individuals had no evidence of IBD or immunological abnormalities at age 11 (Family 6-II:1) and 21 years (Family 7-II:1). *PI4KA* variants identified comprised compound heterozygosity for a missense [p.(Tyr1937Cys), g.21065742T>C, c.5810A>G, gnomAD; absent] alteration in the catalytic domain and frameshift variant [p.(Glu1767fsTer14) in exon 45 of 55, g.21072014_21072015delCA, c.5298_5299delTG, gnomAD; absent] in Family 5-II:1; homozygosity for missense variant [p.(Arg1733Trp), g.21073030G>A, c.5197C>T, gnomAD allele frequency 0.000008 with no homozygotes listed, rs142690596] in Family 6-II:1; and compound heterozygosity for a missense variant [p.(Leu777Pro), g.21119980A>G, c.2330T>C, gnomAD; allele frequency 0.000004 with no homozygotes listed, rs1490645147] and nonsense variant (p.Gln1191Ter in exon 31 of 55, g.21096938G>A, c.3571C>T, gnomAD; absent) in Family 7-II:1. The fifth patient, (Family 8-II:1) was compound heterozygous for one nonsense [p.(Arg566Ter), g.21156289G>A, c.1696C>T, gnomAD allele frequency; 0.0000119 with no homozygotes listed, rs764443420] and one missense variant [p.(Lys1808Thr), g.21068784T>G, c.5423A>C, gnomAD; absent] (Fig. 5A and B). This individual presented at 2 years of age with developmental delay, mild intellectual disability, spasticity and epilepsy that was well controlled with valproic acid. Brain MRI showed diffuse hypomyelination, thin corpus callosum and mild enlargement of subarachnoid spaces (Supplementary Fig. 3). No gastrointestinal or immune abnormalities were noted at 8 years. Notably all missense alterations identified impact evolutionarily invariantly conserved amino acids and were predicted to be damaging (Fig. 5B and C and Supplementary Table 3).

### Molecular modelling provides further insight into the likely molecular basis of the variable clinical outcomes associated with biallelic *PI4KA* variants

**Figure 6:**
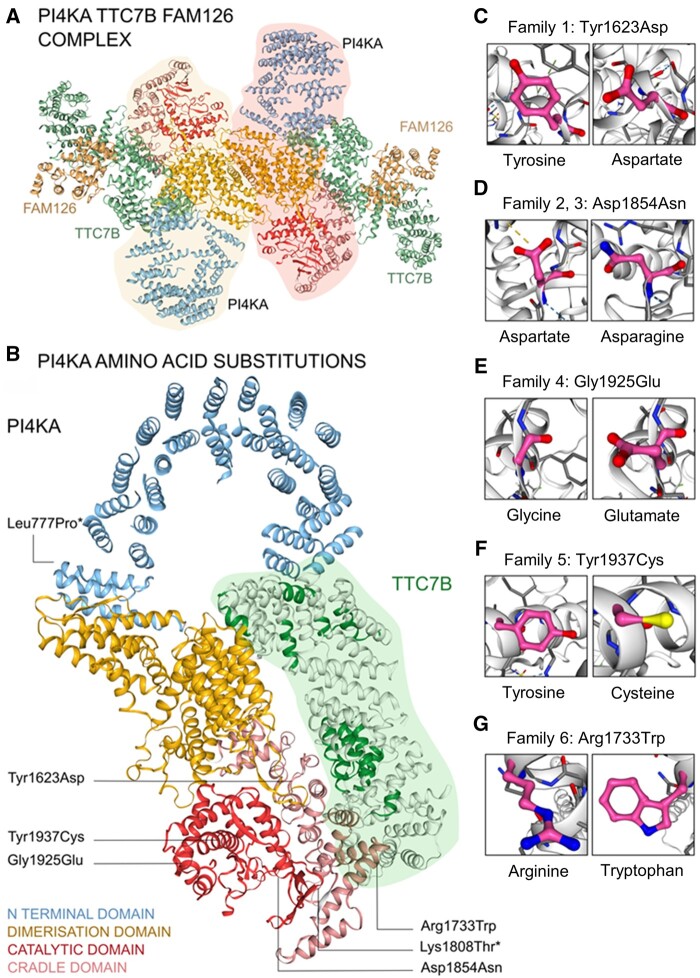
**Molecular modelling of the PI4KIIIα-TTC7(A/B)-FAM126(A/B) complex.** (**A**) The PI4KIIIα-TTC7B-FAM126 homodimer is shown with the PI4KIIIα molecular pair highlighted in pink and yellow. (**B**) PI4KIIIα molecule shown in complex with TTC7B. Four functional regions of PI4KIIIα are shown in different colours: N-terminal alpha solenoid loop (blue), dimerization domain (yellow), cradle domain (pink) and catalytic domain (red). TTC7B is highlighted in green. Highly conserved domains of TTC7B are coloured bright green and correspond to contact points with PI4KIIIα. The positions of PI4KIIIα amino acid substitutions are labelled. The Tyr1623Asp variant is located close to a highly conserved contact point of TTC7. (**C**–**G**) Detailed images of PI4KIIIα amino acid substitutions showing wild-type (*left*) and variant (*right*) amino acids. *Closest possible positions of the Lys1808Thr and Leu777Pro variants are shown. In these regions of the PI4KIIIα molecule, cryo-electron microscopy had insufficient resolution to allow accurate modelling.

It is notable that our genetic studies identified no affected individuals with biallelic variants which are overtly loss of function in nature. This indicates that as in mice, complete loss of PI4KIIIα molecular function is incompatible with life, and that any monogenic disease-associated missense alterations are probably hypomorphic in nature. To provide additional understanding of how candidate *PI4KA* variants may impact PI4KIIIα-TTC7(A/B)-FAM126 complex interactions and function, we mapped the genetic variants identified in our study and performed molecular modelling of the PI4KIIIα-TTC7(A/B)-FAM126(A/B) complexes (Fig. 6). Important functional regions of the PI4KIIIα molecule include an N-terminal domain which forms an alpha solenoid loop, a dimerization domain and a cradle region surrounding the catalytic domain. Three regions of PI4KIIIα are known to interact with TTC7B; the tip of the alpha solenoid loop, the dimerization region and the cradle region.^6^ These PI4KIIIα-TTC7 contact points correspond to highly conserved TTC7B regions.

The p.(Asp1854Asn) PI4KIIIα alteration identified in the previously described foetuses (Family 2^12^) and the child from Family 3 (II:2) is associated with CNS abnormalities, immune system dysfunction, IBD and gastrointestinal pathology consistent with an epithelial organogenesis defect. The Asp1854Asn substitution affects the catalytic domain in line with its reduced PI4KIIIα enzymatic activity, impacting both PI4KIIIα-TTC7A and PI4KIIIα-TTC7B complexes. The p.(Gly1925Glu) and p.(Tyr1937Cys) variants identified in Families 4-II:3 and 5-II:1 affect the catalytic domain and are associated with both CNS abnormalities and IBD. In contrast the Tyr1623Asp substitution identified in the Amish neonates associated with the severe gastrointestinal phenotype of MIA reminiscent of TTC7A deficiency, is located in the cradle region close to the PI4KIIIα-TTC7 molecular interface and distant to the catalytic site, consistent with our finding that the variant displays near normal catalytic activity (Fig. 4D). To investigate the potential outcome of this and the other *PI4KA* gene variants in the context of TTC7A binding and complex formation, we performed homology modelling of TTC7A based on the existing TTC7B structure (Fig. 6). While cryo-electron microscopy structural data of the PI4KIIIα-TTC7A complex is not available, *in silico* modelling of the PI4KIIIα-TTC7A complex revealed probably important and previously unrecognized distinct interaction surfaces for TTC7A and TTC7B. This suggests TTC7A and TTC7B share similar but not identical binding sites, including the region potentially compromised by the Tyr1623Asp alteration, which appears to selectively impair TTC7A binding. The three-dimensional structure of the PI4KIIIα-TTC7B-FAM126 complex has previously been determined by cryo-electron microscopy.^7^ Notably Tyr1623 is located in the cradle region close to a known TTC7B binding site. The p.(Tyr1623Asp) amino acid alteration is likely to alter the electrostatic potential of this molecular region through the substitution of a large aromatic for a smaller non-aromatic side chain. The proximity of Tyr1623 to, but not within, the TTC7 interacting region may explain the differential outcomes on TTC7A versus TTC7B binding, which may each possess overlapping but non-identical PI4KIIIα interacting motifs.

## Discussion

Here we define the phenotypical spectrum in individuals with biallelic *PI4KA* sequence variants. Our findings corroborate PI4KIIIα p.(Asp1854Asn) alteration as a cause of brain abnormalities, and additionally identify immunological and gastrointestinal pathologies related to this substitution. Our studies also identify a *PI4KA* c.4867T>G; p.(Tyr1623Asp) Amish founder variant associated with autosomal recessive MIA, defining only the second known molecular cause of this condition.

Five patients with hypomyelinating leukodystrophy were additionally identified, two of whom also had IBD. Our genetic studies found rare predicted deleterious biallelic *PI4KA* variants as a likely cause of disease, and molecular modelling studies defined the likely disease mechanism as impaired PI4KIIIα catalytic function and/or differential impact on complex formation with TTC7A or TTC7B. Alongside nystagmus and spasticity, brain MRI identified hypomyelination as a feature common to all five affected individuals with a neurological presentation resembling Pelizaeus–Merzbacher disease, the prototype of a hypomyelinating leukodystrophy.^16^

Previously, variants in *FAM126A*, the other PI4KIIIα-TTC7 molecular companion, have been associated with another leukodystrophy; hypomyelination and congenital cataracts, characterized by nystagmus, ataxia and spasticity with/without peripheral neuropathy (leukodystrophy hypomyelinating 5; MIM 610532),^13–15^ similar to the presentation of Families 5–8. In three of these individuals, brain MRI revealed areas of T_1_-hypointensity typical of hypomyelination and congenital cataracts.^25^ The hypomyelinating leukodystrophy associated with variants in both *FAM126A* and *PI4KA* described here, may likely relate to the role of PI4P or its further phosphorylated derivatives, which together with myelin basic protein is required for myelin sheath growth.^14,26-29^ Interestingly, PI4KIIIα has been recently shown to play a pivotal role in myelination of the peripheral nervous system.^29^ Mice with a Schwann cell-specific *Pi4ka* deletion display neuropathy motor symptoms and severe myelination defects, with significantly reduced myelin thickness and lipid content most severely affecting phosphatidylserine and phosphatidylethanolamine, two major myelin components.^29^ Notably, PI4KIIIα has been described as a major factor driving phosphatidylserine transport from the endoplasmic reticulum to the plasma membrane^30^ and hence, affecting phosphatidylserine synthesis.^31^ Nerve conductive studies (performed in two individuals) were mildly abnormal in one, therefore prominent neuropathy does not appear to be part of the phenotypic spectrum of *PI4KA*-related disease. Intriguingly, the affected patient from Family 3 (Individual II:2) homozygous for p.(Asp1854Asn), had immature gyration and cerebellar hypoplasia in addition to hypomyelination and clinical epileptic encephalopathy. This has also been described in severe forms of *RARS1*-associated disease, another hypomyelinating leukodystrophy resembling Pelizaeus–Merzbacher-like disease.^32^

Before our study, the only known genetic cause of MIA involved biallelic variants in PI4KIIIα’s complex binding partner TTC7A, confirming perturbations in PI4KIIIα complex function as a pathomolecular basis of this aspect of disease.^33^^,^^34^ TTC7A has been associated with a wide spectrum of gastrointestinal phenotypes including very early-onset IBD with/without combined immunodeficiency^35–39^ and milder IBDs,^18^^,^^38^^,^^40–43^ mirroring our findings associated with *PI4KA* gene alteration. The bowel histology from the Amish infants, and the previously described foetuses^12^ re-evaluated here, show striking similarities to TTC7A-related bowel disease. These include multiple lumen and luminal deformations, absence of epithelial layer and cell debris, indicating that cell damage has occurred, and a very dense cell infiltrate and reactive lymphocytic infiltrate, indicative of a response towards the epithelial barrier defect. Unfortunately, due to the lethality of the bowel disease in Amish infants, it was not possible to fully evaluate the presence or extent of any neurological clinical outcomes. However, given that neurological findings were seen in all other families described in this study, it seems likely that milder neurological features may have been present initially or ultimately develop should affected individuals survive.

The PI4KIIIα-TTC7-FAM126 molecular complex is responsible for generating the bulk of PI4P at the plasma membrane.^2–5^^,^^14^^,^^24^ Importantly, gastrointestinal phenotypes including epithelial defects and inflammation, have been reported in animal models with phosphatidylinositol metabolism defects (Supplementary Table 4).^44–46^ Homozygous *Pi4ka* knock-out adult mice display intestinal inflammation reminiscent of the inflammatory changes seen in very early-onset IBD (Family 4-II:3) and adult onset IBD (Family 5-II:1) cases here.^10^^,^^11^ This indicates that PI4KIIIα function is required throughout life to maintain normal gastrointestinal function.^10^ In epithelial cells, the asymmetric distribution of phosphoinositides^47^ and Rho family GTPases^48^ has been shown to play a key role in apicobasal polarization, which is essential for epithelial lumen formation.^49–51^ It is thus notable that previous studies revealed disruptions to Rho signalling and polarity, growth and differentiation of intestinal epithelial cells in TTC7A deficiency patient-derived MIA intestinal organoid cultures, again suggesting phosphoinositide metabolism may play a key role in epithelial barrier formation.^38^^,^^40^^,^^52^

Given the mechanistic and phenotypic overlap, the findings relating to TTC7A-associated MIA/IBD are likely applicable to individuals with *PI4KA*-associated gastrointestinal disease. Children with TTC7A deficiency have undergone haematopoietic stem cell transplantation, although bowel inflammation and stenosis have persisted despite successful engraftment suggesting a primary gastrointestinal aetiology.^33^^,^^42^^,^^53^ Consistent with this, the only Amish infant with enough patent bowel at initial surgery to attempt resection and subsequent enteral feeding (Fig. 1A; Family1-X:28) also displayed an ongoing IBD process. This infant underwent two subsequent surgeries due to recurrent small bowel stenosis and novel antral atresia. Although children with similar residual bowel lengths may establish enteral feeding with a fair prognosis, the quality of the remaining bowel and ongoing disease process in *PI4KA* or *TTC7A* associated MIA is likely to have further detrimental effects on morbidity and mortality. Taken together, this indicates that TTC7A or PI4KIIIα-related MIA is a severe, currently untreatable disorder in which surgical interventions only leads to immediate mechanical improvement, without affecting the outcome. Importantly, however, leflunomide was recently identified as a preclinical treatment for TTC7A bowel disease, reducing intestinal narrowing and increasing intestinal survival in zebrafish and patient-derived colonoid cellular models.^54^ As TTC7A is known to regulate PI4KIIIα, drugs identified to treat TTC7A deficient inflammatory bowel phenotypes, including MIA, may also be effective in patients with PI4KIIIα deficiency.

Immunological abnormalities, including combined immunodeficiency and abnormal thymic development are commonly a feature in TTC7A-related gastrointestinal disease.^42^ PI4KIIIα is the predominant producer of PI4P and possibly affects other downstream polyphosphoinositides, notably PI[3,4,5]P3, which act as second messengers activating downstream signalling cascades involved in numerous immune cell functions from cell survival and growth, to cell adhesion and migration.^55–57^ A severe T-cell deficiency and agammaglobulinaemia were noted in one Amish infant (Family 1-X:1) and, while not a predominant feature, mild persistent lymphopenia was also reported in a second (Family 1-X:28). Notably, one child (Family 3-II:2) developed follicular lymphoma atypical for her age and was subsequently found to have persistent lymphopaenia and hypogammaglobulinaemia. As a comprehensive immunological work-up was not possible, we are unable to determine whether the immune system in the other individuals affected by *PI4KA*-related disease is entirely normal, and conclusions regarding the clinical spectrum and significance of the immune defects in the *PI4KA*-related disease spectrum will need to be further explored.

While the catalytic subunit of the PI4KIIIα-TTC7-FAM126-EFR3 complex is encoded by a single gene, each of the non-catalytic subunits is encoded by two genes with different patterns of expression. The function of this complex is clearly central to maintain the normal development and function of multiple different tissue types, but the current lack of comprehensive knowledge regarding the tissue-specific role of each complex renders linking genotypes to the highly variable phenotypic outcomes challenging. However, our data and experimental evidence suggests that complete loss of PI4KIIIα function may be incompatible with life, as supported by the embryonic lethality of *Pi4ka* (PI4KIIIα) knockout in mice,^3^ and that disease-associated missense alterations are therefore probably hypomorphic in nature. Testing the effects of such alterations on the assembly and stability of the PI4KIIIa complex using recombinant proteins or tissue culture systems is extremely challenging, in part due to incomplete knowledge of the precise molecular and cellular roles of the complex. Even if this could be achieved, such alterations may affect a specific developmental process in a tissue and developmental stage-specific manner that may not reveal itself as a defect in a tissue culture setting. These limitations have similarly been noted in attempts at defining phenotype correlations linked with specific *TTC7A* gene alterations associated with the same spectrum of bowel disease as seen in *PI4KA*-related disease, involving IBD and defects of gastrointestinal epithelial organogenesis including MIA.^35^^,^^58^ Therefore, the future generation of mutation-specific *PI4KA* and *TTC7A/B* animal models will be highly informative in defining genotype–phenotype correlations and clinical outcomes associated with these genes. Similarly, the identification of further individuals with *PI4KA* gene alterations is of importance to confirm, as our data indicate, that gene alterations primarily impairing catalytic function are associated with more complex multisystem disease affecting the CNS, with variable gastrointestinal and immune involvement. Our molecular studies and modelling data indicate *PI4KA* alterations that lie outside the catalytic domain, but that disrupt normal PI4KIIIα-TTC7-FAM126-EFR3 complex formation, may result in a predominately organ-specific phenotype if the alteration selectively affects interactions with specific non-catalytic subunits. In this study, we identified a more marked impact of the PI4KIIIα p.(Tyr1623Asp) substitution on PI4KIIIα binding with TTC7A versus TTC7B, probably accounting for the severe MIA in the Amish infants. Conversely, the predominantly neurological phenotype in Families 6 and 7, suggests the missense alterations involving residues 777 and 1733 may impact levels or catalytic activity of PI4KIIIα (for example, by an effect on its folding), or affect PI4KIIIα complex formation with TTC7B more severely than TTC7A (the 1773 variant is close to the TTC7 interface). While the molecular basis of the variable immunological and gastrointestinal phenotype remains unclear, it is important to reiterate that *TTC7A* variants are similarly associated with a broad disease spectrum, and that the absence of robust functional assays has prevented a systematic functional characterization of missense alterations in this gene.^35^^,^^58^ Future studies that improve our understanding of the structure, binding sites and tissue-specific roles of PI4KIIIa complexes containing TTC7A or TTC7B are required to more precisely elucidate the relationship between the location of *TTC7A* and *PI4KA* gene variants and phenotypic outcomes.

We define biallelic *PI4KA* alterations as a new genetic cause of a complex and clinically variable disorder affecting neurological, immunological and intestinal development and function, stemming from selective PI4KIIIα-TTC7 functional impairment. Our findings suggest that all individuals with suspected *PI4KA*-associated disease should be assessed for the neurological, immunological and gastrointestinal presentations of the disorder. Taken together, our data provide a molecular rationale to begin to explain how *PI4KA* gene variants cause different phenotypes, providing fundamental new insights into the varying roles of PI4KIIIα-TTC7A/B-FAM126 complexes in distinct cell types and developmental processes.

## Supplementary Material

awab313_Supplementary_Data

## Abbreviations

IBD: inflammatory bowel disease
MIA: multiple intestinal atresia
PI4KIIIα: phosphatidylinositol 4-kinase alpha
PI4P: phosphatidylinositol 4-phosphate
WES: whole-exome sequencing
WGS: whole genome sequencing

## References

[1] Boura E , Nencka R. Phosphatidylinositol 4-kinases: Function, structure, and inhibition. Exp Cell Res. 2015;337(2):136–145. 26183104 10.1016/j.yexcr.2015.03.028

[2] Balla A , Tuymetova G, Tsiomenko A, Varnai P, Balla T. A plasma membrane pool of phosphatidylinositol 4-phosphate is generated by phosphatidylinositol 4-kinase type-III alpha: Studies with the PH domains of the oxysterol binding protein and FAPP1. Mol Biol Cell. 2005;16(3):1282–1295. 15635101 10.1091/mbc.E04-07-0578 PMC551492

[3] Nakatsu F , Baskin JM, Chung J, et al PtdIns4P synthesis by PI4KIIIalpha at the plasma membrane and its impact on plasma membrane identity. J Cell Biol. 2012;199(6):1003–1016. 23229899 10.1083/jcb.201206095 PMC3518224

[4] Baird D , Stefan C, Audhya A, Weys S, Emr SD. Assembly of the PtdIns 4-kinase Stt4 complex at the plasma membrane requires Ypp1 and Efr3. J Cell Biol. 2008;183(6):1061–1074. 19075114 10.1083/jcb.200804003 PMC2600738

[5] Wu X , Chi RJ, Baskin JM, et al Structural insights into assembly and regulation of the plasma membrane phosphatidylinositol 4-kinase complex. Dev Cell. 2014;28(1):19–29. 24360784 10.1016/j.devcel.2013.11.012 PMC4349574

[6] Lees JA , Zhang Y, Oh MS, et al Architecture of the human PI4KIIIalpha lipid kinase complex. Proc Natl Acad Sci U S A. 2017;114(52):13720–13725. 29229838 10.1073/pnas.1718471115 PMC5748228

[7] Balla T. Phosphoinositides: Tiny lipids with giant impact on cell regulation. Physiol Rev. 2013;93(3):1019–1137. 23899561 10.1152/physrev.00028.2012 PMC3962547

[8] Di Paolo G , De Camilli P. Phosphoinositides in cell regulation and membrane dynamics. Nature. 2006;443(7112):651–657. 17035995 10.1038/nature05185

[9] Baba T , Alvarez-Prats A, Kim YJ, et al Myelination of peripheral nerves is controlled by PI4KB through regulation of Schwann cell Golgi function. Proc Natl Acad Sci U S A. 2020;117(45):28102–28113. 33106410 10.1073/pnas.2007432117 PMC7668188

[10] Bojjireddy N , Botyanszki J, Hammond G, et al Pharmacological and genetic targeting of the PI4KA enzyme reveals its important role in maintaining plasma membrane phosphatidylinositol 4-phosphate and phosphatidylinositol 4,5-bisphosphate levels. J Biol Chem. 2014;289(9):6120–6132. 24415756 10.1074/jbc.M113.531426 PMC3937678

[11] Vaillancourt FH , Brault M, Pilote L, et al Evaluation of phosphatidylinositol-4-kinase IIIalpha as a hepatitis C virus drug target. J Virol. 2012;86(21):11595–11607. 22896614 10.1128/JVI.01320-12 PMC3486294

[12] Pagnamenta AT , Howard MF, Wisniewski E, et al Germline recessive mutations in PI4KA are associated with perisylvian polymicrogyria, cerebellar hypoplasia and arthrogryposis. Hum Mol Genet. 2015;24(13):3732–3741. 25855803 10.1093/hmg/ddv117 PMC4459391

[13] Traverso M , Yuregir OO, Mimouni-Bloch A, et al Hypomyelination and congenital cataract: Identification of novel mutations in two unrelated families. Eur J Paediatr Neurol. 2013;17(1):108–111. 22749724 10.1016/j.ejpn.2012.06.004

[14] Baskin JM , Wu X, Christiano R, et al The leukodystrophy protein FAM126A (hyccin) regulates PtdIns(4)P synthesis at the plasma membrane. Nat Cell Biol. 2016;18(1):132–138. 26571211 10.1038/ncb3271 PMC4689616

[15] Zara F , Biancheri R, Bruno C, et al Deficiency of hyccin, a newly identified membrane protein, causes hypomyelination and congenital cataract. Nat Genet. 2006;38(10):1111–1113. 16951682 10.1038/ng1870

[16] Wolf NI , Ffrench-Constant C, van der Knaap MS. Hypomyelinating leukodystrophies - unravelling myelin biology. Nat Rev Neurol. 2020;17(2):88–103. 33324001 10.1038/s41582-020-00432-1

[17] Miyamoto Y , Torii T, Eguchi T, Nakamura K, Tanoue A, Yamauchi J. Hypomyelinating leukodystrophy-associated missense mutant of FAM126A/hyccin/DRCTNNB1A aggregates in the endoplasmic reticulum. J Clin Neurosci. 2014;21(6):1033–1039. 24417797 10.1016/j.jocn.2013.09.014

[18] Fayard J , Collardeau S, Bertrand Y, et al TTC7A mutation must be considered in patients with repeated intestinal atresia associated with early inflammatory bowel disease: Two new case reports and a literature review. Arch Pediatr. 2018;25(5):334–339. 10.1016/j.arcped.2018.05.006 29921470

[19] Saunders JR , Lehman A, Turvey SE, et al Novel exonic deletions in TTC7A in a newborn with multiple intestinal atresia and combined immunodeficiency. J Clin Immunol. 2019;39(6):616–619. 31342292 10.1007/s10875-019-00669-6

[20] Broome DT , Young A, Torbic H, et al Combined immunodeficiency with inflammatory bowel disease in a patient with TTC7A deficiency. ACG Case Rep J. 2019;6(5):e00061. 31616743 10.14309/crj.0000000000000061 PMC6658069

[21] Stals KL , Wakeling M, Baptista J, et al Diagnosis of lethal or prenatal-onset autosomal recessive disorders by parental exome sequencing. Prenat Diagn. 2018;38(1):33–43. 29096039 10.1002/pd.5175 PMC5836855

[22] Swiss Anabaptist Genealogical Association SAGA database. Accessed 19 May 2021. http://www.saga-omii.org/TNG1112/

[23] Uhlen M , Fagerberg L, Hallstrom BM, et al Proteomics. Tissue-based map of the human proteome. Science (New York, NY). 2015;347(6220):1260419. 10.1126/science.1260419 25613900

[24] Dornan GL , Dalwadi U, Hamelin DJ, Hoffmann RM, Yip CK, Burke JE. Probing the architecture, dynamics, and inhibition of the PI4KIIIalpha/TTC7/FAM126 complex. J Mol Biol. Sep 14 2018;430(18 Pt B):3129–3142. 30031006 10.1016/j.jmb.2018.07.020

[25] Steenweg ME , Vanderver A, Blaser S, et al Magnetic resonance imaging pattern recognition in hypomyelinating disorders. Brain. 2010;133(10):2971–2982. 20881161 10.1093/brain/awq257 PMC3589901

[26] Heller BA , Ghidinelli M, Voelkl J, et al Functionally distinct PI 3-kinase pathways regulate myelination in the peripheral nervous system. J Cell Biol. 2014;204(7):1219–1236. 24687281 10.1083/jcb.201307057 PMC3971744

[27] Maurel P , Salzer JL. Axonal regulation of Schwann cell proliferation and survival and the initial events of myelination requires PI 3-kinase activity. J Neurosci. 2000;20(12):4635–4645. 10844033 10.1523/JNEUROSCI.20-12-04635.2000 PMC6772460

[28] Goebbels S , Oltrogge JH, Kemper R, et al Elevated phosphatidylinositol 3,4,5-trisphosphate in glia triggers cell-autonomous membrane wrapping and myelination. J Neurosci. 2010;30(26):8953–8964. 20592216 10.1523/JNEUROSCI.0219-10.2010 PMC6632897

[29] Alvarez-Prats A , Bjelobaba I, Aldworth Z, et al Schwann-cell-specific deletion of phosphatidylinositol 4-kinase alpha causes aberrant myelination. Cell Rep. 2018;23(10):2881–2890. 29874576 10.1016/j.celrep.2018.05.019 PMC7268203

[30] Chung J , Torta F, Masai K, et al INTRACELLULAR TRANSPORT. PI4P/phosphatidylserine countertransport at ORP5- and ORP8-mediated ER-plasma membrane contacts. Science. 2015;349(6246):428–432. 26206935 10.1126/science.aab1370 PMC4638224

[31] Sohn M , Ivanova P, Brown HA, et al Lenz-Majewski mutations in PTDSS1 affect phosphatidylinositol 4-phosphate metabolism at ER-PM and ER-Golgi junctions. Proc Natl Acad Sci U S A. 2016;113(16):4314–4319. 27044099 10.1073/pnas.1525719113 PMC4843478

[32] Mendes MI , Green LMC, Bertini E, et al RARS1-related hypomyelinating leukodystrophy: Expanding the spectrum. Ann Clin Transl Neurol. 2020;7(1):83–93. 31814314 10.1002/acn3.50960 PMC6952319

[33] Samuels ME , Majewski J, Alirezaie N, et al Exome sequencing identifies mutations in the gene TTC7A in French-Canadian cases with hereditary multiple intestinal atresia. J Med Genet. 2013;50(5):324–329. 23423984 10.1136/jmedgenet-2012-101483 PMC3625823

[34] Guttman FM , Braun P, Garance PH, et al Multiple atresias and a new syndrome of hereditary multiple atresias involving the gastrointestinal tract from stomach to rectum. J Pediatr Surg. 1973;8(5):633–640. 4752999 10.1016/0022-3468(73)90401-6

[35] Jardine S , Dhingani N, Muise AM. TTC7A: Steward of intestinal health. Cell Mol Gastroenterol Hepatol. 2019;7(3):555–570. 30553809 10.1016/j.jcmgh.2018.12.001 PMC6406079

[36] Stenson PD , Ball EV, Mort M, et al Human Gene Mutation Database (HGMD): 2003 update. Hum Mutat. 2003;21(6):577–581. 12754702 10.1002/humu.10212

[37] Celli J. Genetics of gastrointestinal atresias. Eur J Med Genet. 2014;57(8):424–439. 25019371 10.1016/j.ejmg.2014.06.007

[38] Lemoine R , Pachlopnik-Schmid J, Farin HF, et al Immune deficiency-related enteropathy-lymphocytopenia-alopecia syndrome results from tetratricopeptide repeat domain 7A deficiency. J Allergy Clin Immunol. 2014;134(6):1354–1364.e6. 25174867 10.1016/j.jaci.2014.07.019

[39] Uhlig HH , Schwerd T, Koletzko S, et al; COLORS in IBD Study Group and NEOPICS. The diagnostic approach to monogenic very early onset inflammatory bowel disease. Gastroenterology. 2014;147(5):990–1007.e3. 25058236 10.1053/j.gastro.2014.07.023 PMC5376484

[40] Avitzur Y , Guo C, Mastropaolo LA, et al Mutations in tetratricopeptide repeat domain 7A result in a severe form of very early onset inflammatory bowel disease. Gastroenterology. 2014;146(4):1028–1039. 24417819 10.1053/j.gastro.2014.01.015 PMC4002656

[41] Lawless D , Mistry A, Wood PM, et al Bialellic mutations in tetratricopeptide repeat domain 7A (TTC7A) cause common variable immunodeficiency-like phenotype with enteropathy. J Clin Immunol. 2017;37(7):617–622. 28808844 10.1007/s10875-017-0427-1

[42] Fernandez I , Patey N, Marchand V, et al Multiple intestinal atresia with combined immune deficiency related to TTC7A defect is a multiorgan pathology: Study of a French-Canadian-based cohort. Medicine (Baltimore). 2014;93(29):e327. 25546680 10.1097/MD.0000000000000327 PMC4602622

[43] Agarwal NS , Northrop L, Anyane-Yeboa K, Aggarwal VS, Nagy PL, Demirdag YY. Tetratricopeptide repeat domain 7A (TTC7A) mutation in a newborn with multiple intestinal atresia and combined immunodeficiency. J Clin Immunol. 2014;34(6):607–610. 24931897 10.1007/s10875-014-0067-7

[44] Thakur PC , Davison JM, Stuckenholz C, Lu L, Bahary N. Dysregulated phosphatidylinositol signaling promotes endoplasmic-reticulum-stress-mediated intestinal mucosal injury and inflammation in zebrafish. Dis Model Mech. 2014;7(1):93–106. 24135483 10.1242/dmm.012864 PMC3882052

[45] Zhao S , Xia J, Wu X, et al Deficiency in class III PI3-kinase confers postnatal lethality with IBD-like features in zebrafish. Nat Commun. 2018;9(1):2639. 29980668 10.1038/s41467-018-05105-8 PMC6035235

[46] Uno JK , Rao KN, Matsuoka K, et al Altered macrophage function contributes to colitis in mice defective in the phosphoinositide-3 kinase subunit p110delta. Gastroenterology. 2010;139(5):1642–53.e1-6. 20637203 10.1053/j.gastro.2010.07.008 PMC2967619

[47] Martin-Belmonte F , Mostov K. Phosphoinositides control epithelial development. Cell Cycle. 2007;6(16):1957–1961. 17712229 10.4161/cc.6.16.4583

[48] Mack NA , Georgiou M. The interdependence of the Rho GTPases and apicobasal cell polarity. Small GTPases. 2014;5(2):10. 25469537 10.4161/21541248.2014.973768 PMC4601375

[49] Bryant DM , Datta A, Rodríguez-Fraticelli AE, Peränen J, Martín-Belmonte F, Mostov KE. A molecular network for de novo generation of the apical surface and lumen. Nat Cell Biol. 2010;12(11):1035–1045. 20890297 10.1038/ncb2106 PMC2975675

[50] Martin-Belmonte F , Gassama A, Datta A, et al PTEN-mediated apical segregation of phosphoinositides controls epithelial morphogenesis through Cdc42. Cell. 2007;128(2):383–397. 17254974 10.1016/j.cell.2006.11.051 PMC1865103

[51] Shewan A , Eastburn DJ, Mostov K. Phosphoinositides in cell architecture. Cold Spring Harbor Perspect Biol. 2011;3(8):a004796. 10.1101/cshperspect.a004796 PMC3140688 21576256

[52] Bigorgne AE , Farin HF, Lemoine R, et al TTC7A mutations disrupt intestinal epithelial apicobasal polarity. J Clin Invest. 2014;124(1):328–337. 24292712 10.1172/JCI71471 PMC3871247

[53] Kammermeier J , Lucchini G, Pai SY, et al Stem cell transplantation for tetratricopeptide repeat domain 7A deficiency: Long-term follow-up. Blood. 2016;128(9):1306–1308. 27418642 10.1182/blood-2016-01-696385

[54] Jardine S , Anderson S, Babcock S, et al Drug screen identifies leflunomide for treatment of inflammatory bowel disease caused by TTC7A deficiency. Gastroenterology. 2020;158(4):1000–1015. 31743734 10.1053/j.gastro.2019.11.019 PMC7062591

[55] Koyasu S. The role of PI3K in immune cells. Nat Immunol. 2003;4(4):313–319. 12660731 10.1038/ni0403-313

[56] Gambardella L , Vermeren S. Molecular players in neutrophil chemotaxis–focus on PI3K and small GTPases. J Leukoc Biol. 2013;94(4):603–612. 23667166 10.1189/jlb.1112564

[57] Hawkins PT , Stephens LR, Suire S, Wilson M. PI3K signaling in neutrophils. Curr Top Microbiol Immunol. 2010;346:183–202. 20473789 10.1007/82_2010_40

[58] El-Daher MT , Lemale J, Bruneau J, et al Chronic intestinal pseudo-obstruction and lymphoproliferative syndrome as a novel phenotype associated with tetratricopeptide repeat domain 7A deficiency. Front Immunol. 2019;10:2592. 31787977 10.3389/fimmu.2019.02592 PMC6853864

