# Supplementary material for: Biallelic PI4KA variants cause neurological, intestinal and immunological disease
Source: Brain. 2021 Aug 20;144(12):3597–610. doi: 10.1093/brain/awab313 (PMC8719846; doi:10.1093/brain/awab313)
Supplement: awab313_Supplementary_Data [file awab313_supplementary_data.zip › awab313-suppl_data/brain-2021-00168-File011.pdf]

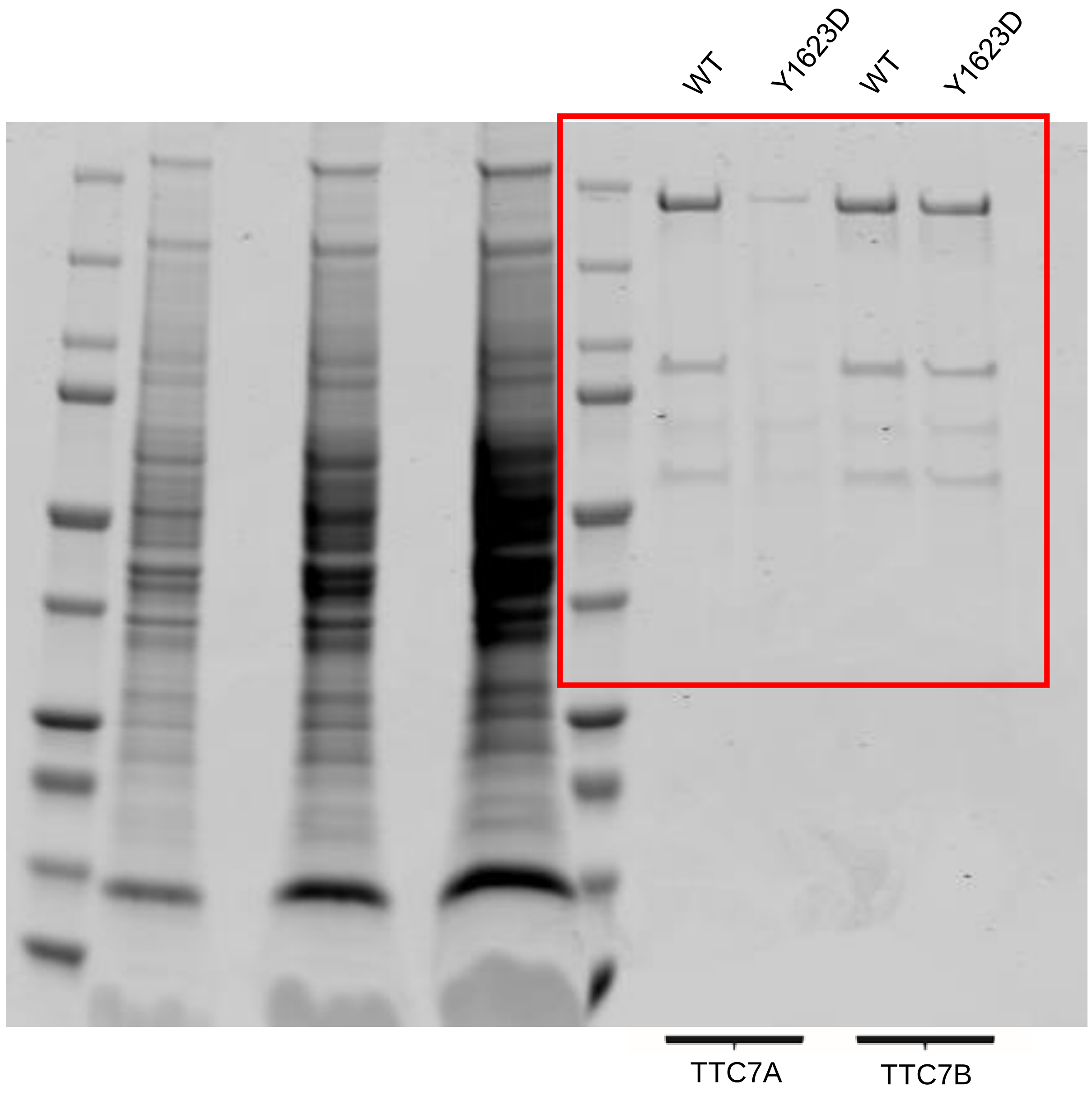

Full unedited gel for Figure 4A; The portion in the red frame was used in figure

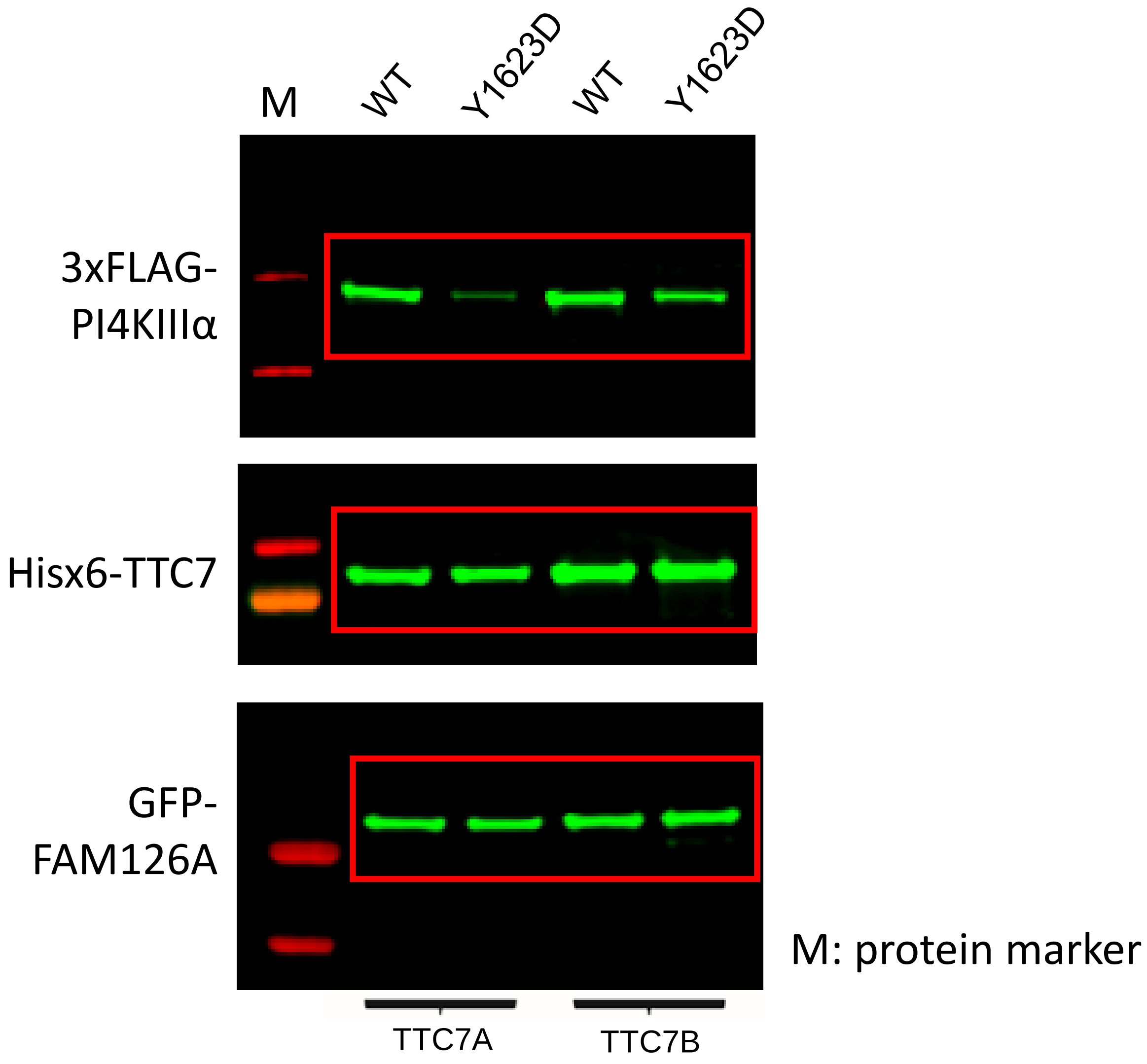

Full unedited gel for Figure 4C: Original western blot.
The portions in the red frames were used in figure

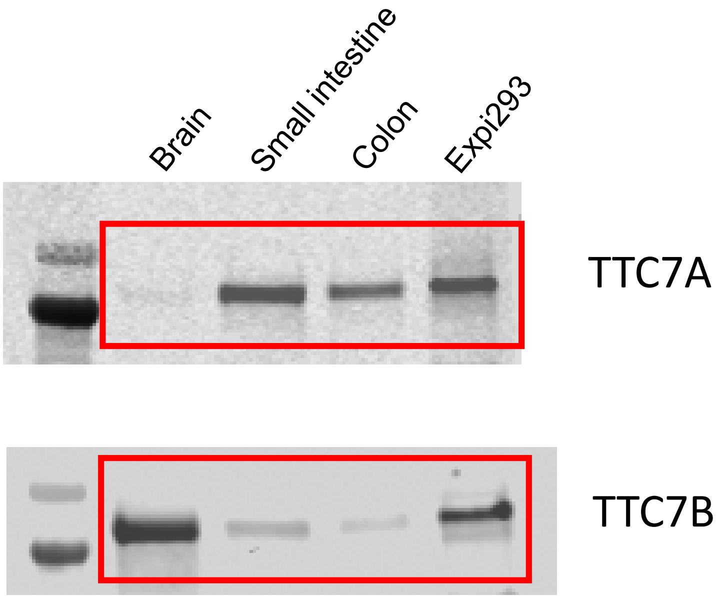

Full unedited gel for Figure 4F: Original western blot.
The portions in the red frames were used in figure
