# Supplementary material for: Biallelic PI4KA variants cause neurological, intestinal and immunological disease
Source: Brain. 2021 Aug 20;144(12):3597–610. doi: 10.1093/brain/awab313 (PMC8719846; doi:10.1093/brain/awab313)
Supplement: awab313_Supplementary_Data [file awab313_supplementary_data.zip › awab313-suppl_data/Salter et al_PI4KA_Supplementary Material_16.11.21_FINAL.docx]

**Biallelic *PI4KA* variants cause neurological, intestinal and immunological disease**

**Supplementary Material**

Claire G. Salter,^1,2,†^ Yiying Cai,^3,4,5,6,†^ Bernice Lo,^7,8,†^ Guy Helman,^9,10,†^ Henry Taylor,^11^ Amber McCartney,^3,4,5,6^ Joseph S. Leslie,^1^ Andrea Accogli,^12^ Federico Zara,^12^ Monica Traverso,^12^ James Fasham,^1,13^ Joshua A. Lees,^4^ Matteo P. Ferla,^14^ Barry A. Chioza,^1^ Olivia Wenger,^15^ Ethan Scott,^15^ Harold E. Cross,^16^ Joanna Crawford,^10^ Ilka Warshawsky,^17^ Matthew Keisling,^17^ Dimitris Agamanolis,^17^ Catherine Ward Melver,^17^ Helen Cox,^18^ Mamoun Elawad,^19^ Tamas Marton,^20^ Matthew N. Wakeling,^1^ Dirk Holzinger,^21^ Stephan Tippelt,^21^ Martin Munteanu,^22^ Deyana Valcheva,^23^ Christin Deal,^24^ Sara Van Meerbeke,^24^ Catherine Walsh Vockley,^25^ Manish J. Butte,^26^ Utkucan Acar,^26^ Marjo S. van der Knaap,^27,28^ G. Christoph Korenke,^29^ Urania Kotzaeridou,^30^ Tamas Balla,^31^ Cas Simons,^9,10^ Holm H. Uhlig,^32,33,34,‡^ Andrew H. Crosby,^1,‡^ Pietro De Camilli,^3,4,5,6,35,‡^ Nicole I. Wolf^27,‡^ and Emma L. Baple^1,13,‡^

^†^**^,‡^These authors contributed equally to this work.**

1. RILD Wellcome Wolfson Centre, University of Exeter Medical School, Exeter, UK
2. Wessex Clinical Genetics Service, Princess Anne Hospital, Southampton, UK
3. Department of Neuroscience, Yale University School of Medicine, New Haven, CT, USA
4. Department of Cell Biology, Yale University School of Medicine, New Haven, CT, USA
5. Program in Cellular Neuroscience Neurodegeneration and Repair, Yale University School of Medicine, New Haven, CT, USA
6. Howard Hughes Medical Institute, Yale University School of Medicine, New Haven, CT, USA
7. Research Branch, Sidra Medicine, Doha, Qatar
8. College of Health and Life Sciences, Hamad Bin Khalifa University, Doha, Qatar
9. Murdoch Children's Research Institute, The Royal Children’s Hospital, Parkville, Melbourne, Australia
10. Institute for Molecular Bioscience, The University of Queensland, Brisbane, Australia
11. Department of surgery and Cancer, Imperial College London, London, UK
12. IRCCS Istituto Giannina Gaslini, 16147 Genova, Italy
13. Peninsula Clinical Genetics Service, Royal Devon and Exeter Hospital, Exeter, UK
14. Wellcome Centre Human Genetics, University of Oxford, Oxford, UK
15. New Leaf Center, Mt. Eaton, OH, USA
16. Department of Ophthalmology, University of Arizona College of Medicine, Tucson, AZ, USA
17. Akron Children’s Hospital, Akron, OH, USA
18. West Midlands Clinical Genetics Service, Birmingham Women’s and Children’s Hospital, Birmingham, UK
19. Department of Gastroenterology, Sidra Medicine, Doha, Qatar
20. West Midlands Perinatal Pathology, Birmingham Women’s and Children’s Hospital, Edgbaston, Birmingham, UK
21. Department of Pediatric Haematology-Oncology, Pediatrics III, University of Duisburg-Essen, Essen, Germany
22. Institute for Human Genetics, University Hospital Essen, University Duisburg-Essen, Essen, Germany
23. Department of Pediatrics, Sana Kliniken Duisburg, Germany
24. Children’s Hospital of Pittsburgh, UPMC, Division of Pediatric Allergy and Immunology, Pittsburgh, USA
25. Children’s Hospital of Pittsburgh, UPMC, Division of Genetic and Genomic Medicine, Pittsburgh, USA
26. Department of Paediatrics, Division of Immunology, Allergy, and Rheumatology, UCLA, Los Angeles, CA, USA
27. Amsterdam Leukodystrophy Center, Department of Child Neurology, Emma Children's Hospital, Amsterdam University Medical Center, VU University Amsterdam and Amsterdam Neuroscience, 1081 HV Amsterdam, The Netherlands
28. Department of Functional Genomics, Centre for Neurogenomics and Cognitive Research, Vrije Universiteit Amsterdam, 1081 HV Amsterdam, The Netherlands
29. Department of Neuropediatrics, University Children's Hospital, Klinikum Oldenburg, 26133 Oldenburg, Germany
30. Department of Child Neurology and Metabolic Medicine, Center for Pediatric and Adolescent Medicine, University Hospital Heidelberg, D-69120 Heidelberg, Germany
31. Section on Molecular Signal Transduction, Eunice Kennedy Shriver National Institute of Child Health and Human Development, National Institutes of Health, Bethesda, MD, USA
32. Translational Gastroenterology Unit, NIHR Oxford Biomedical Research Centre, John Radcliffe Hospital, University of Oxford, Oxfordshire, UK
33. Department of Paediatrics, University of Oxford, Oxfordshire, UK
34. Oxford NIHR Biomedical Research Centre, Oxford, UK
35. Kavli Institute for Neuroscience, Yale University School of Medicine, New Haven, CT, USA

**Contents**

[**Supplementary methods** 4](#_Toc80087832)

[Genetics investigations 4](#_Toc80087833)

[Molecular modelling 5](#_Toc80087834)

[**Supplementary figure 1:** Co-purification of the PI4KIIIα-TTC7-FAM126A complex with EFR3 7](#_Toc80087835)

[**Supplementary figure 2:** Activity assay of the PI4KIIIα-TTC7A-FAM126A and PI4KIIIα-TTC7B-FAM126A complexes 8](#_Toc80087836)

[**Supplementary figure 3:** Neuroimaging findings in individuals with candidate biallelic *PI4KA* variants 9](#_Toc80087837)

[**Supplementary table 1:** A comparison of clinical findings of affected individuals homozygous or compound heterozygous for *PI4KA* variants 11](#_Toc80087838)

[**Supplementary table 2:**  Detailed surgical and bowel histology findings in individuals with *PI4KA* variants 16](#_Toc80087839)

[**Supplementary immunophenotyping data 1 (Family 1-X:1)** 17](#_Toc80087840)

[**Supplementary immunophenotyping data 2 (Family 3-II:2)** 18](#_Toc80087841)

[**Supplementary table 3:** Variants identified from whole exome or genome sequencing 20](#_Toc80087842)

[**Supplementary table 4:** Gastrointestinal phenotypes in animal models with disruption of phosphatidylinositol metabolism 25](#_Toc80087843)

[**References** 26](#_Toc80087844)

# **Supplementary methods**

## **Genetics investigations**

In Family 1 (Amish), DNA was extracted from paraffin embedded bowel tissue samples obtained from four affected neonates (X:2, X:8, X:26, X:28), peripheral blood from X:1, and peripheral blood/or buccal samples obtained from each parent and 14 unaffected siblings, using standard techniques. Whole exome sequencing (WES) was performed at the University of Exeter on DNA from IX:3, 4, 5, 6, 7 and 8, and X:28, as previously described, using the SureSelect Human All Exon V6 (Agilent Technologies) exome enrichment kit on an Illumina NextSeq500 sequencer.^1^ Reads were aligned to the human genome reference sequence (hg19) using Burrows-Wheeler Aligner (BWA)-MEM (v0.7.17), mate pairs were fixed and duplicates removed using Picard (v2.15). InDel realignment and base quality recalibration were performed using GATK (v3.7.0). SNVs and InDels were detected using GATK HaplotypeCaller and annotated using Alamut batch (v1.8). Read depth was determined for the whole exome through our in-house pipeline. Copy number variants were detected using both ExomeDepth (<https://cran.r-project.org/web/packages/ExomeDepth/vignettes/ExomeDepth-vignette.pdf> and <https://github.com/vplagnol/ExomeDepth>) and SavvyCNV ([https://www.biorxiv.org/content/10.1101/617605v1](https://eur03.safelinks.protection.outlook.com/?url=https%3A%2F%2Fwww.biorxiv.org%2Fcontent%2F10.1101%2F617605v1&data=02%7C01%7CC.Salter%40exeter.ac.uk%7C7ecd4e5ca5e746ff9ad708d795aaca39%7C912a5d77fb984eeeaf321334d8f04a53%7C0%7C0%7C637142433085693844&sdata=d8RhBs83RYfbjNwlYa6QrtUeKl7%2ByLd5dRBSkdIuc9M%3D&reserved=0) and [https://github.com/rdemolgen/SavvySuite](https://eur03.safelinks.protection.outlook.com/?url=https%3A%2F%2Fgithub.com%2Frdemolgen%2FSavvySuite&data=02%7C01%7CC.Salter%40exeter.ac.uk%7C7ecd4e5ca5e746ff9ad708d795aaca39%7C912a5d77fb984eeeaf321334d8f04a53%7C0%7C0%7C637142433085703833&sdata=V3PuKGaNobdxYSFTuZ1uqjvTDVR9bP21fkmZVG6HsjY%3D&reserved=0)). Variants with <5 reads, a frequency of >1% in gnomAD (V2.1.1) and/or in-house databases were excluded. *De novo*, homozygous or compound heterozygous variants present in exons or within ±6 nucleotides in the intron were evaluated in X:28, which identified the homozygous variant in *PI4KA*. Exome sequencing and haplotype analysis in IX:3, 4, 5, 6, 7 and 8 was utilised to exclude variants in *TTC7A*, with all coding and splice junctions adequately covered at 10x (mean depth 116 in all six parental samples and 86 in proband; coverage at 20x was 98% in the parental samples and 95% in proband). Any remaining compound heterozygous and homozygous variants present genome wide in X:28 were filtered against the exome sequencing data from IX: 3, 4, 5, and 6, taking into consideration coverage.

Whole Genome Sequencing (WGS) and trio data analysis was performed on DNA from affected infant Family 1-X:1 and both parents (IX:1 and 2) using PCR free Illumina sequencing libraries run on a HiSeq2500 sequencing (to 45X coverage), Bcl files were aligned to hg19/GRCH build 37 and nucleotide variants called with DRAGEN (Edico Genome). Structural variants (SVs) were called using the union of four algorithms (BreakSeq, CNVnator, Delly, and Manta) and annotated to determine affected genes and regulatory regions and were compared to public and internal databases to determine minor allele frequency and filter common, or false positive SVs. The filtering steps followed for this trio were similar to those described for Family 1-X:28 and identified the same homozygous *PI4KA* variant.

Family 3 were investigated as part of routine clinical care, trio WES was performed at Praxis für Humangenetik Tübingen, on DNA from the patient and both parents. The encoding and adjacent intronic region, as well as the known pathogenic and likely pathogenic intronic variants were enriched with a hybridization- in-solution technology and sequenced on the Illumina platform. Sequencing data was processed with Illumina bcl2fastq2 and reads were aligned to the human genome reference sequence (hg19) using Burrows-Wheeler Aligner. Only variants with a minor allele frequency of less than 0.01 within coding regions and flanked intronic regions (+/-8 nucleotides) were reported. Known pathogenic variants seen in HGMD were additionally analysed up to a minor allele frequency of less than 0.05 and in +/-30 nucleotide flanking regions.

For Family 4, DNA from the proband and parents was whole genome sequenced to an average coverage of 30 reads per base on the Illumina HiSeq X system in the Genomics core facility at Sidra Medicine. Reads were aligned to the hg19 human reference sequence using BWA. Single nucleotide variant and indel calling were performed using GATK HaplotypeCaller. For variant quality filtering, GATK Variant Quality Score Recalibration (VQSR) was used. SnpEff/SnpSift was used to annotate the VCF files. Only non-synonymous, nonsense, indel, and splicing junction variants (+/- 6 nucleotides) were considered. Filtering was based on variant quality, recessive or *de novo* mode of inheritance, allele frequency (<0.005; 1000 Genomes Project, The Genome Aggregation Database), and predicted outcome (SIFT, PolyPhen, MutationTaster) to prioritize high quality missense and likely pathogenic variants.

Families 5, 6 and 7 were investigated as part of an on-going study by the Amsterdam Database of Unclassified Leukoencephalopathies (hypomyelination cohort) to unravel the genetic cause of unclassified leukodystrophies. Trio WGS was performed and analysed as previously described on all nuclear family members.^2^ Sequencing was performed using 2x150-nucleotide paired-end reads on an Illumina X10 by Illumina Cambridge Ltd. Alignment of reads to hg19 was performed using the BWAr. Variant calling for WGS was performed using GATK HaplotypeCaller v3.7. Variant annotation was performed using SnpEff v4.3m and a custom pipeline was used for variant filtration and prioritisation using population allele frequency, predicted impact on gene function, and *de novo* and recessive models of inheritance.

For Family 8, trio WES was performed at the University of Exeter as previously described on DNA from the affected individual and both parents, using Twist Human Core Exome targeting.^1^ Read alignment (BWA-MEM (v0.7.17)) was performed, mate-pairs fixed and duplicates removed (Picard v2.15.0). InDel realignment/base quality recalibration (GATK v3.7.0), single-nucleotide variant (SNV)/InDel detection (GATK HaplotypeCaller) and annotation (Alamut Batch v1.8 or v1.10) were performed and read depth (in-house pipeline) calculated. Copy number variants were detected using both ExomeDepth and SavvyCNV. The trio variants filtering steps were the same as those described for the affected Amish infant and his parents from Family 1 (X:28).

Genetic investigation methodology for Family 2 can be found in the original publication by Pagnamenta et al. in 2015.^3^

In all cases the variants were then assessed for clinical correlation with the affected individual’s phenotype. Unique primers were designed and utilised for amplification and bidirectional dideoxy sequencing of all *PI4KA* gene variants identified.

## **Molecular modelling**

The three dimensional structure of the PI4KIIIα, TTC7, and FAM126 complex was visualised using the web based application MichelaNGLo^4, 5^ and the protein databank (PDB)^6, 7^ cryo-electron microscopy structural entry 6BQ1.^8^ The PI4KIIIα mutations were introduced to the model and further analysed using MichelaNGLo’s Variant Effect on Structure (VENUS) tool, which utilised structural data from UniProt.^9^ Mutations were mapped within the PI4KIIIα molecule and the macromolecular complex. Mutation position was interpreted relative to four previously identified functional regions of the PI4KIIIα structure^8^: the N terminal region which forms an alpha solenoid loop (residues 1-956), a dimerization domain (residues 957-1536), a cradle region (residues 1537-1787), and the C terminal catalytic domain (residues 1788-2085). The effect of the mutation was predicted by considering the properties of the substituted amino acids including changes in size, shape, polarity, charge, and rigidity. A clash was identified when the distance between residues was one or more Angstroms less than the sum of the van der Waals radii.

The degree of conservation of amino acid residues within TTC7B was assessed using the ConSurf web server (http://consurf.tau.ac.il).^10, 11^ Normalised conservation scores for each amino acid were calculated. Regions of 10 amino acids or longer with a mean conservation score of <-0.75 were labelled as conserved and mapped to the TTC7B molecule using MichelaNGLo.

Two in silico models of TTC7A were assessed. One was generated via the Phyre2 server^12^ and the other obtained via SwissModel.^13^ Threading of the region across from the cradle region of PI4KIIIα was incorrect in the Phyre2 model, so the SwissModel was used. Cryo-electron microscopy structure 6BQ1^8^ was energy minimised in Pyrosetta^14^ against its density map. The TTC7B in the model was replaced by the SwissModel TTC7A and energy minimised first with fixed backbone, then against the density map and then unconstrained. Difference in free energy was calculated with PyRosetta by mutating the pose and minimising 10 Å around its Cα, and then scoring with the ref2015 score function. Interface strength was calculated using the InterfaceAnalyzerMover mover of PyRosetta and the 10 Å neighbourhood deviation was calculated using the all_atom_rmsd score. Missing loops were added with PyRosetta, but not used for scoring. A model of ATP and phosphatidylinositol was made by docking ATP constrained by the common pharmacophores with the GSKA1 inhibitor and binding of ATP in PDB:5I0N.  A butyryl-tailed PI was docked constrained as if about to attack to the ATP.

# **Supplementary figure 1: Co-purification of the PI4KIIIα-TTC7-FAM126A complex with EFR3**

pcDNA3.1-3×FLAG-PI4KIIIα, pCMV-AN-His-GFP-EFR3B, pCMV6-AN-His-TTC7A or pCMV6-AN-His-TTC7B, and EGFP-FAM126A were co-expressed in Expi293 cells. The complexes were pulled down by anti-FLAG M2 resin, eluted by 3xFLAG tag peptide, subjected to SDS-PAGE and visualized by Coomassie Blue. Both TTC7A (left) and TTC7B (right) form a complex with wildtype PI4KIIIα, EFR3 and FAM126. The band indicated by an asterisk is a chaperone.





# **Supplementary figure 2: Activity assay of the PI4KIIIα-TTC7A-FAM126A and PI4KIIIα-TTC7B-FAM126A complexes**

5nM of each purified complex (PI4KIIIα/TTC7A/FAM126A and PI4KIIIα/TTC7B/FAM126A) was used in the assay (n=3). The reaction was performed at room temperature for 10 min.

**
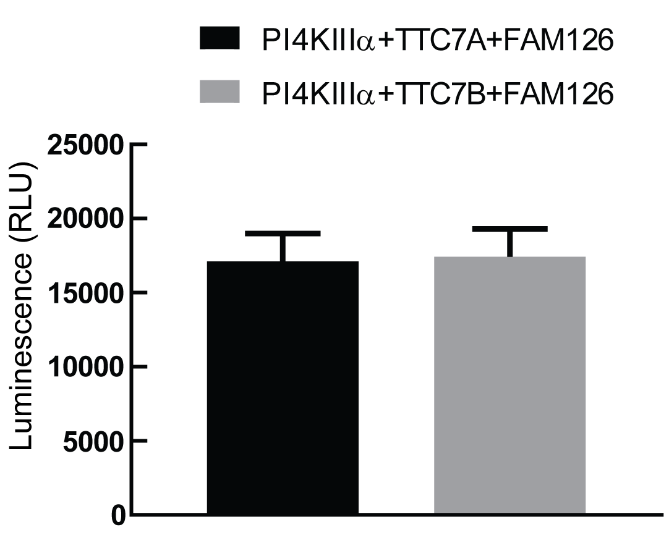
**

# **Supplementary figure 3: Neuroimaging findings in individuals with candidate biallelic *PI4KA* variants**


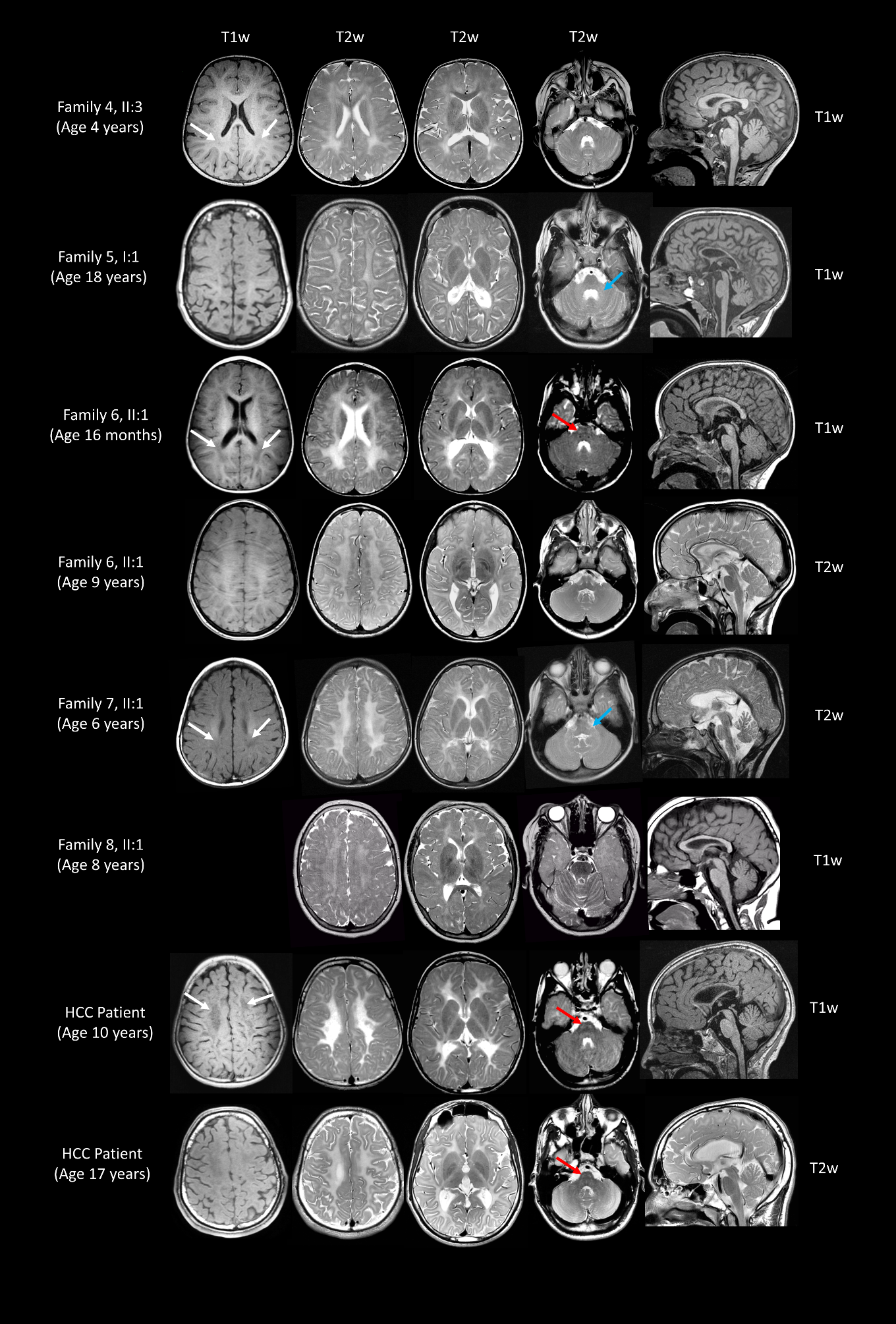


Axial and sagittal MR images of probands from Families 4-8 and, for comparison, MRIs of a patient with hypomyelination and congenital cataracts (HCC) due to a homozygous *FAM126A* variant. All patients show a T2 hyperintense signal of the supratentorial white matter. In the younger patients (Families 4-II:3, 6-II:1, 7-II:1 and HCC patient at age 10 y), this signal is relatively high, with areas of relatively hypointense T1 signal (white arrows). In later MRIs, the white matter signal becomes more homogenous and less T2 hyperintense. In the brain stem, pyramidal tracts show a T2 hyperintense signal in some patients (red arrows). The cerebellar white matter is affected in some patients (blue arrows), with a hyperintense T2 signal. The sagittal images show a thin corpus callosum in the older patients due to loss of white matter, and only very mild cerebellar atrophy.

# **Supplementary table 1: A comparison of clinical findings of affected individuals homozygous or compound heterozygous for *PI4KA* variants**

| **Family (ID)** | **Family 1(X:1)** | **Family 1 (X:2)** | **Family 1 (X:8)** | **Family 1 (X:26)** | **Family 1 (X:28)** | **Family 2 (II:1)** | **Family 2 (II:2)** | **Family 2 (II:5)** |
| --- | --- | --- | --- | --- | --- | --- | --- | --- |
| ***PI4KA* variants**  **(NM_058004.3;NP_477352)** | p.(Tyr1623Asp)/  p.(Tyr1623Asp) | p.(Tyr1623Asp)/  p.(Tyr1623Asp) | p.(Tyr1623Asp)/  p.(Tyr1623Asp) | p.(Tyr1623Asp)/  p.(Tyr1623Asp) | p.(Tyr1623Asp)/  p.(Tyr1623Asp) | p.(Arg796*) / p.(Asp1854Asn) | p.(Arg796*) / p.(Asp1854Asn) | p.(Arg796*) / p.(Asp1854Asn) |
| **Ethnicity** | Amish | Amish | Amish | Amish | Amish | European | European | European |
| **Gender** | Male | Female | Male | Male | Male | Female | Female | Female |
| **Gestation** | 43 wks | 37 wks | Term | Not known | 35+5 wks | TOP 34 wks | TOP 28 wks | TOP 16 wks |
| **Birth weight (centile)**  **Birth OFC (centile)** | 3100g (25th)  Not known | 1980g (2nd)  Not known | 2436g (0.4th)  Not known | 2000g (NA)  Not known | 2808g (75th)  Not known | 2328g (55th)  Not known | 1404g (98th)  Not known | 138g (expect 90g)  Not known |
| **Current age** | Died at 3 wks | Died at 3 days | Died at 10 days | Died in first week | Died at 1 month | NA | NA | NA |
| **NEUROLOGICAL FEATURES** | | | | | | | | |
| **Age of onset of**  **neurological symptoms** | No symptoms observed | No symptoms observed | No symptoms observed | No symptoms observed | No symptoms observed | Antenatal | Antenatal | Antenatal |
| **Signs at presentation** | NA | NA | NA | NA | NA | Bilateral talipes equinovarus | Bilateral talipes equinovarus, flexed knees | Bilateral talipes equinovarus, flexed knees and left wrist |
| **Seizures** | ✘ | ✘ | ✘ | ✘ | ✘ | NA | NA | NA |
| **NEUROIMAGING** | | | | | | | | |
| **Polymicrogyria** | ✘ | Not known | Not known | Not known | Not known | Bilateral asymmetric perisylvian | Bilateral perisylvian | Bilateral perisylvian |
| **Cerebellar and/or**  **brainstem anomalies** | ✘ | Not known | Not known | Not known | Not known | Cerebellar hypoplasia,  small pons | Cerebellar hypoplasia, dysplastic dentate nuclei | Cerebellar dysplasia, dysplastic dentate and olivary nuclei |
| **Leukodystrophy** | Not known | Not known | Not known | Not known | Not known | Not known | Not known | Not known |
| **Other features** | Normal cranial ultrasound. Further imaging not performed | Not known | Not known | Not known | Not known | Asymmetric cerebral ventriculomegaly | Not known | Not known |
| **GASTROINTESTINAL FEATURES** | | | | | | | | |
| **Age of onset of gastrointestinal symptoms** | Antenatal,  gestation unknown | Antenatal,  gestation unknown | Antenatal,  gestation unknown | Antenatal,  gestation unknown | Antenatal,  gestation unknown | Antenatal,  gestation unknown | Antenatal,  gestation unknown | Antenatal,  gestation unknown |
| **Multiple intestinal atresia** | ✔ | ✔ | ✔ | ✔ | ✔ | ✘ | ✘ | Abnormality of epithelial organogenesis |
| **Inflammatory bowel disease** | Not known | ✔ | ✔ | ✔ | ✔ | Not known | Not known | Not known |
| **IMMUNOLOGICAL FEATURES** | | | | | | | | |
| **Immunodeficiency** | Severe lymphopenia, hypogamma-globulinaemia | Not known | Normal full blood count day one of life | Normal full blood count day one of life | Intermittent mild lymphopenia, one episode of marked lymphopenia | NA | NA | NA |
| **OTHER CLINICAL FEATURES** | | | | | | | | |
|  | Normal thymus ultrasound | None noted | None noted | None noted | None noted | Bilateral renal pelviectasis,  mild lung hypoplasia, micrognathia | Unilateral renal pelviectasis, borderline lung hypoplasia, dolicocephaly,  micrognathia,  small tongue and absent uvula,  overlapping 2nd and 5th fingers | Micrognathia,  dolicocephaly |

| **Family (ID)** | **Family 3 (II:2)** | **Family 4 (II:3)** | **Family 5 (II:1)** | **Family 6 (II:1)** | **Family 7 (II:1)** | **Family 8 (II:1)** |
| --- | --- | --- | --- | --- | --- | --- |
| ***PI4KA* variants**  **(NM_058004.3; NP_477352.3)** | p.(Asp1854Asn)/ p.(Asp1854Asn) | p.(Gly1925Glu)/  p.(Arg2022GlnfsTer36) | p.(Tyr1937Cys)/  p.(Glu1767SerfsTer14) | p.(Arg1733Trp)/  p.(Arg1733Trp) | p.(Gln1191*)/  p.(Leu777Pro) | p.(Arg566*)/  p.(Lys1808Thr) |
| **Ethnicity** | Turkish | Indian | German | Turkish | German | Italy |
| **Gender** | Female | Female | Female | Male | Female | Female |
| **Gestation** | Term | 37 wks | Term | Term | Term | Term |
| **Birth weight (centile)**  **Birth OFC (centile)** | 3650g (75^th^)  30 cm (<0.4^th^) | 3170 g (75th)  33cm (50^th^) | 2800 g (9-25th)  34cm (50^th^) | 3300 g (25-50th)  Not known | 2440 g (2nd)  Not known | 3450g (50th)  Not known |
| **Current age** | 13 yrs | 5 yrs | 24 yrs | 11 yrs | 21 yrs | 8 yrs |
| **Current height; weight; OFC (centile)** | 116cm (<0.4^th^), 18.4kg (<0.4^th^), 48cm (<0.1^st^) | 100 cm (2^nd^); 18.25kg (50^th^); 48cm (0.1^st^) | 154cm (7^th^); 40kg (<0.4^th^); 52cm (0.6^th^) | 130cm (2^nd^); 27.7kg (9^th^); 51.8cm (3^rd^) | 164cm (50^th^); 57.7kg (50^th^); 54cm (14^th^) | Not known |
| **NEUROLOGICAL FEATURES** | | | | | | |
| **Age of onset of neurological symptoms** | First day of life | 2 yrs | First week of life | 6 months | 6 months | 2 yrs |
| **Signs at presentation** | Refractory seizures | Global developmental delay | Irritability,  intermittent feeding problems, hypotonia | Delayed motor development | Nystagmus,  delayed motor development | Hand tremor, axial hypotonia,  left equinus foot deformity |
| **Disease progression** | Stable | Very slow developmental progress,  progressive spastic diplegia | Very slow developmental  progress,  progressive limb spasticity  lower>upper | Progressive limb spasticity  lower>upper,  mild ataxia | Pyramidal and cerebellar signs, developmental regression from  age 15 yrs | Progressive limb spasticity  lower>upper |
| **Developmental delay** | Global developmental delay | Global developmental delay | Global developmental delay | Global developmental delay | Global developmental delay | Global developmental delay |
| **Intellectual disability** | Severe intellectual disability | Mild intellectual disability | Moderate intellectual disability | Moderate intellectual disability | Moderate intellectual disability | Mild intellectual disability |
| **Behavioural abnormalities** | Sleep disorder and restlessness | ✘ | ✘ | ✘ | Depressive mood,  anxiety disorder,  refusal to eat. | ✘ |
| **Gross motor** | Rolling, unable to sit or stand, no head control. Truncal hypotonia, limb spasticity | Weight bears, not crawling, unable to maintain 4 point stance | Able to sit with support, unable to walk | Crawling and standing with support (age 15 months), walks with a retrowalker (6 yrs), scissoring of legs,  climbs onto sofa | Sitting without support  (age 5 yrs), walking with support (age 6 yrs), loss of walking with support  (age 12 yrs) | Never walked independently, few steps with support (8 yrs) |
| **Fine motor** | Severe impairment of fine motor function, grasping movements not possible | Fine tremors that affects coordination | Manipulates objects with difficulty | Manipulates objects with difficulty | Manipulates objects with difficulty | Manipulates objects with difficulty |
| **Speech and Language** | No speech or vocalisations. Communicates via talker. Laughs and cries | First words ~18 months. Several words, understands simple commands.  No dysarthria | Speaks several words, better receptive language skills | Speak 2-3 word sentences, understands 2 languages.  Mild dysarthria | Speaks several words,  better receptive language skills.  Dysarthria | Mild language delay |
| **Dysphagia** | Mild | Drinks or eats from a spoon. Unable to chew | Yes, gastrostomy at 21 yrs | Mild, eats with a spoon | Progressive dysphagia. Gastrostomy at 16 yrs | ✘ |
| **Vision** | Optic atrophy, convergent strabismus | Normal | Decreased visual acuity, nystagmus | Normal vision, saccadic pursuit, fine pendular nystagmus and gaze-evoked nystagmus | Decreased visual acuity, pendular nystagmus | Mild horizontal nystagmus,  visual evoked potentials showed abnormal retinal-cortical conductions |
| **Head titubation** | ✘ | ✘ | ✘ | Mild | ✔ | Not known |
| **Hearing** | Hearing loss  (has hearing aid) | Normal | Normal | Normal | Normal | Normal |
| **Cerebellar signs** | Not known | ✘ | Ataxia with intention tremor and dysmetria | Mild intention tremor and dysmetria, mild ataxia | Intention tremor and dysmetria | Hand tremor,  axial hypotonia |
| **Dystonia** | ✔ | Dystonic hand movements | ✘ | ✘ | ✘ | ✘ |
| **Seizures** | Epileptic encephalopathy, West-syndrome during infancy. Tonic, myoclonic and atypical absence seizures | ✘ | Age 7 yrs: GTC,  controlled with Sultiam; | ✘ | Age 6 yrs: GTC,  controlled with Sultiam.  Relapse age 13 yrs: GTC and stimulus-induced myoclonus, controlled with Levetiracetam and  Valproic acid | Age 3 yrs: recurrent GTC, controlled with Depakin.  Seizure free by 8 yrs |
| **EEG findings** | 2021: Non-specific general background changes, no focal findings, multifocal irregular sharp wave activity right fronto-precentral and left temporal | No epileptic discharges | Central and temporal spike-wave complexes | NA | Age 13 yrs: bilateral centroparietal sharp waves.  Age 18 yrs: diffuse mild slowing | Multifocal epileptic activities in the parieto-temporal-occipital regions, more pronounced in the right hemisphere |
| **Nerve conduction studies** | Not performed | Not performed | No results available | Age 1 yr: normal  Age 9 yrs: very mild abnormalities compatible with axonal neuropathy | Age 7 and 16 yrs: Normal | Not performed |
| **EXAMINATION** | | | | | | |
| **Age on examination** | 12 yrs | 4 yrs | 23 yrs | 10 yrs | 16 yrs | 8 yrs |
| **Upper limbs** | | | | | | |
| **Muscle tone** | Elevated | Normal | Mildly elevated | Mildly elevated | Elevated | Elevated |
| **Tendon reflexes** | Increased | Normal | Normal | Increased | Increased | Increased |
| **Sensory function** | Not known | No evident abnormalities | No evident abnormalities | No evident abnormalities | No evident abnormalities | No evident abnormalities |
| **Lower limbs** | | | | | | |
| **Muscle tone** | Elevated (spasticity) | Elevated (spasticity) | Elevated (spasticity) | Elevated (spasticity) | Elevated (spasticity) | Elevated (spasticity) |
| **Tendon reflexes** | Increased | Increased | Increased | Increased | Increased | Increased |
| **Plantar responses** | Upgoing bilaterally | Upgoing bilaterally | Upgoing bilaterally | Upgoing bilaterally | Upgoing bilaterally | Upgoing bilaterally |
| **Sensory function** | Not known | No evident abnormalities | No evident abnormalities | No evident abnormalities | No evident abnormalities | No evident abnormalities |
| **NEUROIMAGING** | | | | | | |
| **Polymicrogyria** | Immature gyral pattern | ✘ | ✘ | ✘ | ✘ | ✘ |
| **Cerebellar and/or brainstem anomalies** | Cerebellar hypoplasia and cerebellar atrophy after status epilepticus | ✘ | Mild atrophy of pons and medulla oblongata,  mild cerebellar atrophy | ✘ | Mild atrophy of pons and medulla oblongata,  mild cerebellar atrophy | ✘ |
| **Leukodystrophy** | Diffuse T2-elevation of white matter signal in the entire supratentorial white matter  Thin corpus callosum | Profound hypomyelination.  Myelin present in the posterior limbs of the internal capsules, lateral thalami, and within the corpus callosum and brainstem, but the rest of the brain appears hypo-myelinated.  No progression in myelination seen in serial imaging with advancing age | Diffuse T2-elevation of white matter signal in the entire supratentorial white matter with loss of parieto-occipital white matter  Severe thinning of the corpus callosum | Diffuse T2-elevation of white matter signal in the entire supratentorial white matter with some loss of parieto-occipital white matter  Some T1-hypointense white matter areas    Slight thinning of the corpus callosum | Diffuse T2-elevation of white matter signal in the entire supratentorial white matter with loss of parieto-occipital white matter  Some T1-hypointense white matter areas  Severe thinning of the corpus callosum | Diffuse T2 hyperintensity in the bilateral cortical regions |
| **Other features** | At age 3 yrs asymmetric supratentorial atrophy (left >> right) | ✘ | Progressive supratentorial atrophy | ✘ | Progressive supratentorial atrophy | Mild corpus callosum hypoplasia and mild enlargement of subarachnoid spaces |
| **GASTROINTESTINAL FEATURES** | | | | | | |
| **Age of onset of gastrointestinal symptoms** | 3 yrs (anaemia) | 6 wks | 19 yrs | NA | NA | NA |
| **Multiple intestinal atresia** | ✘ | ✘ | ✘ | ✘ | ✘ | ✘ |
| **Inflammatory bowel disease** | Persistent iron deficiency anaemia, raised faecal calprotectin | Colitis,  no small bowel involvement | Pancolitis with proctocolectomy and terminal ileum resection | ✘ | ✘ | ✘ |
| **IMMUNOLOGICAL FEATURES** | | | | | | |
| **Immunodeficiency** | B cell lymphopenia, hypogammaglobulinaemia, moderate lymphopenia with marked reduced B- and NK cells. Mildly reduced activated and absent CD4+ T cells,  Expansion of senescent CD57+ CD8+ T cells,  Elevated transitional and CD21 low B cells as well as reduced naive B cells.  Follicular non-Hodgkin lymphoma (non-paediatric) grade 3a | Autoimmune enteropathy | ✘ | ✘ | ✘ | ✘ |
| **OTHER CLINICAL FEATURES** | | | | | | |
|  | Chronic lung disease; interstitial lung emphysema and bronchiectasis,  scoliosis, hyperthyroidism,  poor sleep | Rectovaginal fistula | Kyphosis, delayed dentition, increased bleeding tendency, prolonged menstruation required blood transfusions,  Goldnetz™-endometrial ablation performed aged 24 yrs | Pronounced kyphosis | Pronounced kyphosis | Left equinus foot deformity |

**Abbreviations**: wks, weeks; yrs, years; g, grams; TOP, termination of pregnancy; OFC, occipitofrontal circumference; cm, centimetres; NA, not available; (✔), indicates presence of a feature in an affected subject; (✘), indicates absence of a feature in an affected subject; GTC, Generalised tonic clonic seizures.

Centiles calculated from UK-WHO data.

# **Supplementary table 2: Detailed surgical and bowel histology findings in individuals with *PI4KA* variants**

| **ID** | **SURGICAL FINDINGS** | **HISTOLOGY** |
| --- | --- | --- |
| **Family 1**  **X:1** | Oesophagus mildly dilated, pyloric atresia.  Massively dilated duodenum, small bowel diminutive with large sections of atresia interspersed with 1-2 cm islands of normal bowel. Sections of large bowel appeared string like, rectum tiny. Small bowel malrotation. | Samples not retained. |
| **Family 1**  **X:2** | Distal duodenal atresia.  Pyloric atresia.  Multiple small bowel and colonic atresia’s with no viable lumen.  Dilatation of common bile duct. | *Duodenum:* patent.  Bowel otherwise ‘string-like’ with occasional patent sections, mucinous material in lumen. Muscularis propria, submucosa and autonomic plexus normal.  *Jejunum:* Several tiny lumina, some with mucosal necrosis, foamy macrophages. Lumina show neutrophils and calcification. Septa across dilated sections.  *Colon:* No lumen. Central core of connective tissue.  *Liver:* Cholestasis; extensive extramedullary haematopoiesis; signs of extrahepatic biliary obstruction. |
| **Family 1**  **X:8** | Multiple intermittent atresia’s from jejunum to anus, with total atresia of small intestine and ‘string-like’ colon.  *Patent areas*: Bubbles of mucus containing bowel.  *Atretic areas*: cord-like, no lumen. | *Jejunum*: Complete muscular wall with normal ganglion cells. No gross lumen. Multiple, central, small luminal spaces lined by columnar epithelium with underlying lymphoid tissue and surrounded by muscularis mucosa. No granulation tissue/ scarring. |
| **Family 1**  **IX:26** | Pyloric atresia.  Multiple sections of atresia through small intestine and colon; Small areas of patent bowel then long fibrous atresia. | Alternating sections of lumen patency and occlusion.  *Bowel wall*: normal muscle and ganglion cells.  *Lumen:* compromised or obliterated, associated with mineralisation. |
| **Family 1**  **IX:28** | Multiple recurrent atretic sections from small intestine to anus; 120cm proximal ileum patent but dilated.  Microcolon with multiple atresias; longest patent section 7.5cm.  Malrotation and volvulus, no acute ischaemic change.  Three surgical procedures (over 3 weeks) required for ongoing ileal stenosis and new antral atresia. | *Ileum:* Multiple distinct small lumen, lined by mucosa and muscularis mucosa (sieve-like). Areas of mucosal atrophy and acute neutrophilic inflammation. Focal collections of mucosal and submucosal histiocytes with calcified material.  Muscularis propria and ganglion cell layer normal.  *Colon*: Areas of complete lumen obliteration. Focal mucosal atrophy and neutrophilic inflammation. Widely dilated sections with mucosal flattening and areas of increased mucosal eosinophils.  Subsequent resection: Ileum mucosa absent with highly mineralised granulation tissue. Rare reactive regenerating glands seen. Mineralisation extends to submucosa with foreign body giant cell reaction. Colon mucosa absent with chronic inflammation and fibrous proliferation in submucosal regions. |
| **Family 2**  **II:5** | No gross external anomalies noted on post mortem. No stenosis or atresia’s seen. | Ballooned epithelial cysts |
| **Family 4**  **II:3** | Active colitis without small bowel involvement | Oesophagus normal. Stomach and duodenum chronic, non-specific inflammation |
| **Family 5**  **II:1** | Proctocolectomy age 21 years, followed by resection of the terminal ileum (severe stenosis and inflammation, thickened wall (1 cm)). Delayed wound healing, improving after prednisolone treatment. | Chronic inflammation with regenerating mucosa, intermittent ischemic pattern with band-like necrosis. Mild backwash ileitis. Second operation: severe transmural inflammation. |

# **Supplementary immunophenotyping data 1 (Family 1-X:1)**

Immune evaluation

| **Thymic immigrant test** | **Day of life 3** | **Day of life 18** | **Normal range** |
| --- | --- | --- | --- |
| CD3+/CD4+ | 90% | 90% | 35-64% |
| CD3+/CD45RA+ | 84% | 63% |  |
| CD4+/CD45RA+ | 86% | 57% | 64-95% |
| CD4+/CD45RA+/CD62L+ | 85% | 72% | 61-94% |

| **Mitogens test** | **Day of life 3** | **Day of life 10** | **Normal range** |
| --- | --- | --- | --- |
| Viability | 64.5% | 47% | > 75% |
| PWM CD45 | 5.3% | 5.6% | > 4.5% |
| PWM CD3 | 13.9% | 25.5% | > 3.5% |
| PWM CD19 | 1.0% | - | >3.9% |
| PHA CD45 | 17.2% | 26.3% | > 49.9% |
| PHA CD3 | 39.9% | 65.4% | >58.5% |

| **Lymphocyte subsets** | **Day of life 3** | **Day of life 18** | **Normal range** |
| --- | --- | --- | --- |
| Total CD3 | 307 | 237 | 2,500-5,500/cumm |
| Total CD4 | 274 | 208 | 1,600-4,000/cumm |
| Total CD8 | 15 | 5 | 560-1700/cumm |
| Total CD19 | 114 | 67 | 300-2,000/cumm |
| Total NK | 84 | 76 | 170-1,100/cumm |
| CD4/CD8 | 17.8 | 41.17 | 1.7-3.1/cumm |

Immunoglobulins measured on Day of Life 3:

IgA < 2 mg/dL (1.3-53 mg/dL)

IgE < 2 KU/L (0.08-6.12 KU/mL)

IgG 997 mg/dL (normal 251-906 mg/dL)

IgM < 10 mg/dL (20-87 mg/dL)

Immunology laboratory summary:

Severe T cell lymphopenia affecting CD8+ T cells more than CD4+ T cells. Appropriate numbers of naïve CD4+ T cells arguing against maternal engraftment. Low B cells and low NK cells. Evidence of agammaglobulinaemia, presuming the IgG is strictly maternal at this measurement on day 3 of life. Normal lymphocyte response to PWM, except for CD19 B cells which had a decreased response. Decreased lymphocyte response to PHA mitogen.

TRECs screening- Day of life 2: Normal

Thymic ultrasound- Day of life 19:

The thyroid gland appears normal. Thymus tissue is seen in the anterior superior mediastinum and appears normal. The calibre of the chest are poorly evaluated.
Impression: Normal appearing thymus.

# **Supplementary immunophenotyping data 2 (Family 3-II:2)**

Bone marrow aspiration age 10yrs: megakaryopoesis shifted to the left and myelopoesis with normal maturation. Eosinophilia. No sign of myelodysplastic syndrome.

Bone marrow biopsy age 10yrs: normal granulopoesis, erythropoesis shifted to the left. Eosinophilia. No sign of myelodysplastic syndrome or haematological disease.

Lymph node biopsy age 10yrs: Follicular lymphoma grade 3A with bcl6-translocation (no MAP2K1 mutation).

Leukocyte differentiation age 10 years:

Leukocytes 7515 N/μl (4500-13000), Granulocytes 5486 N/μl (1800-8000), Lymphocytes 1503 N/μl (1200-5800), Monocytes (CD14+) 526 N/μl (50-800), B-cells (CD19+) - 30 N/μl (100-1160), T-cells (CD3+) 1398 N/μl (708-3792), T-cells (CD4+) 902 N/μl (410-3190), T-cells (CD8+) 406 N/μl (290-2204), NK-cells (CD56+)

Antibody response to previous vaccination:

Diphtheria: IgG - 0. 05 U/ml0 (0.1- 999), Tetanus: IgG - 0. 05 U/ml (0.5- 999), Pneumococci: EIA 3 negative mg

Immunoglobulins age 10 years:

IgA-Serum 2.5 g/l (0.58 – 2.9)

IgG-Serum 5.3 g/l (7.1 – 15.6)

IgM-Serum <0.08 g/l (0.66 – 2.5)

IgE-Serum 49.5 IU/ml (1.9 – 170)

Lymphocyte differentiation:

Moderate lymphopenia with marked reduced B- and NK cells. Mildly reduced activated and absent CD4+ T cells. Expansion of senescent CD57+ CD8+ T cells.

|  | **Patient values** | | **Normal values 12-18 years** | |
| --- | --- | --- | --- | --- |
| **Cell population** | **Percentage** | **Cells/μl** | **Percentage** | **Cells/μl** |
| Leukocyte count |  | 5290 |  | 4400-8100 |
| Lymphocytes | 18.9 | 1119 |  | 1400-3300 |
| CD3+ T Cells | 94.42 | 1056 | 56-84 | 1000-2200 |
| CD4+ of CD3 | 61.77 | 653 | 55-62 | 530-1300 |
| CD8+ of CD3 | 31.88 | 337 | 32-42 | 330-920 |
| CD4/CD8 ratio | 1.94 |  | 1.2-1.7 |  |
| TCRgd+ of CD3 T cells | 4.36 |  | 2-12 |  |
| DNT of TCRab+/CD3+ | 2.38 |  | <2.5 |  |
| B cells | 2.44 | 27 | 6-23 | 110-570 |
| NK cells | 2.78 | 31 | 3-22 | 70-480 |
| HLADR+ of CD4+ T cells | 17.91 | 117 | 4-11 | 30-100 |
| HLADR+ of CD8+ T cells | 19.69 | 66 | 5-25 | 30-180 |
| Naïve CD4+ (CD4+/CD45RA+) | 3.26 | 21 | 33-66 | 230-770 |
| Memory CD4+ (CD4+/CD45RO+) | 95.33 | 622 | 18-38 | 240-700 |
| CD57+ of CD8 T cells | 40.52 | 136 | 13-18 | 81-157 |
| CD27+/CD28+ of CD4 | 87.94 |  | 37-97 |  |
| CD27-/CD28- of CD4 | 0.43 |  | 0.004-5.8 |  |
| CD27+/CD28+ of CD8 | 57.78 |  | 20-95 |  |
| CD27-/CD28- of CD8 | 28.01 |  | 9-65 |  |

B cell differentiation:

Analysis restricted due to low levels of observed B cells and difficult differentiation. Elevated transitional and CD21 low B cells as well as naive B cells.

|  | **Patient values** | **Normal values (10-16 years)** |
| --- | --- | --- |
| **Cell population** | **Percentage** | **Percentage** |
| CD19+ B cells of lymphocytes | 2.1 | 7-24 |
| IgM++CD38++ transitional B cells | 42.40 | 1-25 |
| IgD+CD27- naïve B cells | 17.14 | 49-100 |
| IgD+CD27+ IgM-memory of B cells | 3.78 | 2-28 |
| IgD-CD27+ memory of B cells | 2.52 | 1-43 |
| IgA+CD27+ memory B cells | 0.44 |  |
| IgA+CD27- atypical IgG+ memory B cells | 0.82 |  |
| IgG+CD27+ memory B cells | 0.46 |  |
| IgG+CD27- atypical IgG+ memory B cells | 2.08 |  |
| IgM+/-CD38++ plasmablasts of B cells | 1.28 |  |
| CD21-CD38- of B cells | 30.46 | 1-11 |
| IgD-IgM+CD27+ of B cells (IgM only) | 1.57 | 0.5-7 |

# **Supplementary table 3: Variants identified from whole exome or genome sequencing**

| **Family** | **Gene** | **Zygosity** | | **GRChr37:g.** | **c.** | **p.** | **Pop Freq** | ***In silico* predictions** | | | **Reasons for excluding variant** |
| --- | --- | --- | --- | --- | --- | --- | --- | --- | --- | --- | --- |
|  |  |  |  |  |  |  |  | CADD | Polyphen | SIFT |  |
| **1** | **PI4KA** | **Hom** | **Chr22:**  **g.21081592A>C** | | **NM_058004.3:**  **c.4867T>G** | **NP_477352.3: p.Tyr1623Asp** | **0.000008** | **31** | **probably damaging** | **damaging** | **N/A** |
|  | No additional variants identified after co-segregation and comparison with population specific database | | | | | | | | | | |
|  | | | | | | | | | | | |
| **2** | **PI4KA** | **C Het** | **Chr22:**  **g.21119924G>A** | | **NM_058004.3:**  **c.2386C>T** | **NP_477352.3: p.Arg796Ter** | **0.000012** | **N/A** | **N/A** | **N/A** | **N/A** |
|  | **PI4KA** | **C Het** | **Chr22:**  **g.21067580C>T** | | **NM_058004.3:**  **c.5560G>A** | **NP_477352.3: p.Asp1854Asn** | **0.00002** | **32** | **probably damaging** | **damaging** |  |
|  | See original publication; Pagnamenta et al., 2015^3^ | | | | | | | | | | |
|  | |  | | | | | | | | | |
| **3** | **PI4KA** | **Hom** | | **Chr22:**  **g.21067580C>T** | **NM_058004.3:**  **c.5560G>A** | **NP_477352.3: p.Asp1854Asn** | **0.00002** | **32** | **probably damaging** | **damaging** | **N/A** |
|  | 1.7Mb del (BUB1, ACOXL, BCL2L11, ANAPC1, MERTK, TMEM87B, FBLN7, ZC3H8, ZC3H6, RGPD8) | Het, maternally inherited | | Chr2:g.111395541-113090055del | N/A | N/A | N/A | N/A | N/A | N/A | Recognised CNV: Neurosusceptibility locus with reduced penetrance^15-17^. Inherited from unaffected mother. At most, may partially contribute to developmental delay. |
|  | | | | | | | | | | | |
| **4** | **PI4KA** | **C Het** | | **Chr22:**  **g.21065005delC** | **NM_058004.3:**  **c.6065delG** | **NP_477352.3: p.Arg2022GlnfsTer36** | **0** | **N/A** | **N/A** | **N/A** | **N/A** |
|  | **PI4KA** | **C Het** | | **Chr22:**  **g.21066802C>T** | **NM_058004.3:**  **c.5774G>A** | **NP_477352.3: p.Gly1925Glu** | **0** | **31** | **probably damaging** | **damaging** |  |
|  | HDLBP | *De novo* | | Chr2:  g.242169568G>GT | NM_001320965.3:  c.3590dup | NP_001307894.1:  p.Tyr1197Ter | 0 | N/A | N/A | N/A | High density lipoprotein-binding protein, likely function in cholesterol removal. Minimal neurological expression. |
|  | B4GALT4 | *De novo* | | Chr3:  g.118931037T>C | NM_003778.4:  c.*359A>G  ENST00000471675.1:  c.169A>G | ENSP00000417527.1:  p.Ile57Val | 0 | 6.107 |  | damaging | Responsible for the synthesis of complex-type N-linked oligosaccharide. Minimal neurological expression. |
|  | KIAA1958 | *De novo* | | Ch9:  g.115336514G>A | NM_001287036.2:  c.154G>A | NP_001273965.1:  p.Ala52Thr | 0 | 27.3 | probably damaging | damaging | Uncharacterised protein. Low neurological expression. |
|  | MEF2A | *De novo* | | Chr15:g.100252709CCAGCAGCAGCAGCAGCAG>C | NM_001352617.2:  c.1250_1267del | NP_001339546.1:  p.Gln417_Gln422del | 0 | N/A | N/A | N/A | Phenotype: Autosomal dominant coronary artery disease (OMIM 608320) |
|  | HLA-DRB1 | *De novo* | | Chr6:  g.32549402C>CT | NM_002124.4:  c.583_584insA | NP_002115.2:  p.Arg195GlnfsTer28 | 0.008955 |  |  |  | Phenotype: Major histocompatibility complex. Links to multiple sclerosis and sarcoidosis susceptibility |
|  | RP1L1 | C Het | | Chr8:  g.10465210T>G and Chr8:  g.10469746G>A | NM_178857.6:  c.6398A>C  And  NM_178857.6:  c.1862C>T | NP_849188.4:  p.Glu2133Ala  And  NP_849188.4:  p.Ser621Leu | 0.000016,  0.000029 | 0.194,  7.666 | probably damaging, tolerated | tolerated, tolerated | Phenotype: Occult macular dystrophy (OMIM 613587) and  retinitis pigmentosa (OMIM 618826) |
|  | RP1L1 | C Het | | Chr8:  g.10465830C>G | NM_178857.6:  c.5778G>C | NP_849188.4:  p.Glu1926Asp | 0 | 12.06 | damaging | tolerated |  |
|  | |  | | | | | | | | | |
| **5** | **PI4KA** | **C Het** | | **Chr22:**  **g.21065742T>C** | **NM_058004.3:**  **c.5810A>G** | **NP_477352.3: p.Tyr1937Cys** | **0** | **25.9** | **probably damaging** | **damaging** | **N/A** |
|  | **PI4KA** | **C Het** | | **Chr22:g.21072014_21072015delCA** | **NM_058004.3:**  **c.5298_5299delTG** | **NP_477352.3: p.Glu1767fsTer14** | **0** | **N/A** | **N/A** | **N/A** |  |
|  | SPTAN1 | C Het | | Chr9: g.131377921A>G | NM_001130438.2:  c.5159A>G | NP_001123910.1:  p.Lys1720Arg | 0.000008 | 26.1 | benign | tolerated | Phenotype: Developmental and epileptic encephalopathy 5 (OMIM 613477) |
|  | SPTAN1 | C Het | | Chr9: g.131395481C>T | NM_001130438.2:  c.7309-7C>T | N/A | N/A | N/A | N/A | N/A |  |
|  | TCOF1 | *De novo* | | Chr5: g.149753839G>C | NM_001008657.2:  c.973G>C | NP_001008657.1:  p.Ala325Pro | 0 | 0.090 | benign | tolerated | Phenotype: Treacher Collins syndrome 1  (OMIM 154500) |
|  | |  | | | | | | | | | |
| **6** | **PI4KA** | **Hom** | | **Chr22:**  **g.21073030G>A** | **NM_058004.3:**  **c.5197C>T** | **NP_477352.3: p.Arg1733Trp** | **0.000008** | **31** | **probably damaging** | **damaging** | **N/A** |
|  | NDUFAF6 | Hom | | Chr8: g.96047790A>G | NM_152416.2:  c.406A>G | NP_689629.2:  p.Ile136Val | 0.000004 | 12.71 | benign | tolerated | Phenotype: Mitochondrial complex I deficiency, nuclear type 17 (OMIM 618239). Diagnosis very unlikely due to neuroimaging findings.  Fanconi renotubular syndrome 5 (OMIM 618913) |
|  | DIS3L2 | C Het | | Chr2: g.233075119G>A | NM_152383.4:  c.1204+4G>A | N/A | 0.000016 | N/A | N/A | N/A | Phenotype: Perlman syndrome (OMIM 267000) |
|  | DIS3L2 | C Het | | Chr2: g.233199163G>A | NM_152383.4:  c.2243G>A | NP_689596.4:  p.Arg748His | 0.000029 | 21.5 | benign | damaging |  |
|  | CASR | Hom | | Chr3: g.122003578A>G | NM_001178065.1:  c.2807A>G | NP_001171536.2:  p.Gln936Arg | 0.000096 | 16.47 | benign | tolerated | Phenotype: Hypocalcaemia (OMIM 601198, 145980);  Hyperparathyroidism, neonatal (OMIM 239200) |
|  | SLC26A1 | Hom | | Chr4: g.982761T>A | NM_022042.3:  c.1966A>T | NP_071325.2:  p.Ile656Phe | 0 | 0.398 | benign | damaging | Phenotype: ?Nephrolithiasis, calcium oxalate (OMIM 167030). Gene disruption in mouse model- kidney stones and susceptibility to paracetamol induced liver damage |
|  | NEK9 | C Het | | Chr14: g.75570707G>GA | NM_033116.4: c.1576-8dupT | N/A | 0.000581 | N/A | N/A | N/A | Phenotype: Lethal congenital contracture syndrome 10 (OMIM 617022);  Phenotype: ?Arthrogryposis, Perthes disease, and upward gaze palsy (OMIM 614262) |
|  | NEK9 | C Het | | Chr14: g.75573306A>T | NM_033116.4:  c.1427T>A | NP_001316166.1:  p.Phe476Tyr | 0.000004 | 23.6 | possibly damaging | tolerated |  |
|  | NEK9 | C Het | | Chr14: g.75570707G>GA | NM_033116.4: c.1576-8dupT | N/A | 0.000581 | N/A | N/A | N/A |  |
|  | NEK9 | C Het | | Chr14: g.75573307A>T | NM_033116.4:  c.1426T>A | NP_001316166.1:  p.Phe476Tyr | 0.000004 | 23.9 | possibly damaging | tolerated |  |
|  | PQBP1 | Hemi | | ChrX: g.48755603A>G | ENST00000456306.1: c.3A>G  NM_001032381.2:  c.-18-172A>G | ENSP00000393013.1: p.Ile2Val | 0.000061 | 10.11 | unknown | tolerated | Phenotype: Renpenning syndrome (OMIM 309500). Four hemizygotes in gnomAD |
|  | MNDA | *De novo* | | Chr1: g.158817582T>C | NM_002432.1:  c.1052T>C | NP_002423.1:  p.Val351Ala | 0 | 22.4 | probably damaging | damaging | May act as a transcriptional activator/repressor in the myeloid lineage. Role in the granulocyte/monocyte cell-specific response to interferon. No neurological expression. |
|  | HAUS6 | *De novo* | | Chr9: g.19058467C>A | NM_017645.4:  c.2298G>T | NP_060115.3:  p.Lys766Asn | 0 | 10.5 | benign | tolerated | Contributes to mitotic spindle assembly, maintenance of centrosome integrity and completion of cytokinesis. Low neurological expression. *In silico* benign. KO in mouse model embryologically lethal. |
|  | IQSEC3 | *De novo* | | Chr12: g.284102CG>C | NM_001170738.1:  c.3453delG | NP_001164209.1:  p.Pro1153HisfsTer66 | 0 | N/A | N/A | N/A | Acts as a guanine nucleotide exchange factor for ARF1. Biallelic variants possibly linked to neurodevelopmental disease and fetal akinesia (PMIDs 31130284, 32451403) |
|  | EP400 | *De novo* | | Chr12: g.132537969CAGCAG>C | NM_015409.4:  c.7556_7560delAGCAG | NP_056224.3:  p.Gln2519ProfsTer61 | 0 | N/A | N/A | N/A | Component of the NuA4 histone acetyltransferase complex. Multiple heterozygous LOF variants throughout gene in population databases |
|  | CLEC4G | *De novo* | | Chr19: g.7797778G> GAAAAAAAAAA | ENST00000599020.1:  c.9-13_9-4dup  TTTTTTTTTT | N/A | 0 | N/A | N/A | N/A | Expressed liver and lymph nodes. Acts as receptor for certain viruses. Predicted variant impact and location indicates unlikely to be pathogenic |
|  | |  | | | | | | | | | |
| **7** | **PI4KA** | **C Het** | | **Chr22:**  **g.21119980A>G** | **NM_058004.3:**  **c.2330T>C** | **NP_477352.3: p.Leu777Pro** | **0.000004** | **32** | **probably damaging** | **damaging** | **N/A** |
|  | **PI4KA** | **C Het** | | **Chr22:**  **g.21096938G>A** | **NM_058004.3:**  **c.3571C>T** | **NP_477352.3: p.Gln1191Ter** | **0** | **N/A** | **N/A** | **N/A** |  |
|  | TTN | C Het | | Chr2: g.179419792G>A | NM_001267550.1:  c.88394C>T | NP_001254479.2:  p.Ser29465Phe | 0.003140 | 23.5 | unknown | tolerated | Phenotype: Cardiomyopathy, muscular dystrophy (OMIM 613765,608807,603689,611705,600334) |
|  | TTN | C Het | | Chr2: g.179606393T>C | NM_001267550.1:  c.11567A>G | NP_001254479.2:  p.Asn3856Ser | 0.000014 | 21.8 | unknown | tolerated |  |
|  | TTN | C Het | | Chr2: g.179606393T>C | NM_001267550.1:  c.11567A>G | NP_001254479.2:  p.Asn3856Ser | 0.000014 | 21.8 | unknown | tolerated |  |
|  | TTN | C Het | | Chr2: g.179642515A>G | NM_001267550.1:  c.4396T>C | NP_001254479.2:  p.Phe1466Leu | 0.000687 | 22.4 | unknown | tolerated |  |
|  | VPS13A | C Het | | Chr9: g.79862319C>A | NM_033305.2:  c.2037+8C>A | N/A | 0.004341 | N/A | N/A | N/A | Phenotype: Choreoacanthocytosis (OMIM 200150).  Six homozygotes for splice variant and one homozygote for missense variant in GnomAD |
|  | VPS13A | C Het | | Chr9: g.79931219A>G | NM_033305.2:  c.4760A>G | NP_150648.2:  p.Tyr1587Cys | 0.004644 | 13.22 | benign | tolerated |  |
|  | MYH6 | Hom | | Chr14: g.23862870C>T | NM_002471.2:  c.2928+5G>A | N/A | 0.001750 | N/A | N/A | N/A | Phenotype: Cardiomyopathy (OMIM 613252, 613251);  Atrial septal defect (OMIM 614089) |
|  | GP1BA | *De novo* | | Chr17:  g.4836485C>T | NM_000173.5:  c.586C>T | NP_000164.5:  p.Gln196Ter | 0.000008 | N/A | N/A | N/A | Phenotype: Bernard-Soulier syndrome, type A2 (dominant) (OMIM 153670)  Von Willebrand disease, platelet-type (OMIM 177820) |
|  | | | | | | | | | | | |
| **8** | **PI4KA** | **C Het** | | **Chr22:**  **g.21156289G>A** | **NM_058004.3:**  **c.1696C>T** | **NP_477352.3: p.Arg566Ter** | **0.000012** | **N/A** | **N/A** | **N/A** | **N/A** |
|  | **PI4KA** | **C Het** | | **Chr22:**  **g.21068784T>G** | **NM_058004.3:**  **c.5423A>C** | **NP_477352.3: p.Lys1808Thr** | **0** | **26.7** | **possibly damaging** | **damaging** |  |
|  | USH2A | C Het | | Chr1:  g.215901623C>T | NM_206933.2:  c.11815G>A | NM_206933.2:  p.Glu3939Lys | 0.000465 | 16.77 | possibly damaging | deleterious | Phenotype: Usher syndrome, type 2A (OMIM 276901)  Retinitis pigmentosa 39 (OMIM 613809) |
|  | USH2A | C Het | | Chr1:  g.215807600_215808400del | N/A | N/A | N/A | N/A | N/A | N/A |  |
|  | PIGZ | C Het | | Chr3:  g.196675362G>C | NM_025163.4:  c.406C>G | NM_025163.4:  p.Leu136Val | 0 | 13.97 | benign | tolerated | Phenotype: Associated with central serous chorioretinopathy |
|  | PIGZ | C Het | | Chr3:  g.196675397G>A | NM_025163.4:  c.371C>T | NM_025163.4:  p.Ala124Val | 0.004337 | 0.001 | benign | tolerated |  |
|  | MSH6 | C Het | | Chr2:  g.48028072A>C | NM_000179.3:  c.2950A>C | NM_000179.3:  p.Asn984His | 0.000022 | 23.9 | possibly damaging | deleterious | Phenotype: Mismatch repair cancer syndrome 3 (OMIM 619097) |
|  | MSH6 | C Het | | Chr2: g.48030835_48030838del | NM_000179.3:  c.3438+11_3438+14del | N/A | 0.000216 | N/A | N/A | N/A |  |
|  | ARFGEF3 | C Het | | Chr6:  g.138564113C>A | NM_020340.5:  c.568C>A | NM_020340.5:  p.Gln190Lys | 0.000004 | 20.7 | benign | tolerated | Phenotype: One link to neurological condition but no leukodystrophy  PMID 27431290 |
|  | ARFGEF3 | C Het | | Chr6:  g.138608228G>A | NM_020340.5:  c.2803G>A | NM_020340.5:  p.Ala935Thr | 0.000036 | 28.8 | probably damaging | deleterious |  |
|  | AHCTF1 | C Het | | Chr1:  g.247076606C>T | NM_001323342.2:  c.484G>A | NM_001323342.2:  p.Val162Ile | 0.000028 | 18.09 | benign | tolerated | Required for the assembly of a functional nuclear pore complex (NPC) on the surface of chromosomes as nuclei form at the end of mitosis. Low neurological expression*, in silico* benign. |
|  | AHCTF1 | C Het | | Chr1:  g.247013516C>T | NM_001323342.2:  c.5792G>A | NM_001323342.2:  p.Arg1931His | 0.000048 | 14.17 | benign | tolerated |  |
|  | FMO2 | C Het | | Chr1:  g.171174762G>C | NM_001460.5:  c.1172G>C | NM_001460.5:  p.Arg391Thr | 0.003511 | 27.1 | probably damaging | deleterious | No neurological expression. 10 homozygotes for Arg391Thr in gnomAD, second variant synonymous. |
|  | FMO2 | C Het | | Chr1:  g.171165889C>T | NM_001460.5:  c.423C>T | NM_001460.5:  p.Asp141= | 0.000092 | N/A | N/A | N/A |  |
|  | PXDN | C Het | | Chr2:  g.1651996C>G | NM_012293.3:  c.3556G>C | NM_012293.3:  p.Glu1186Gln | 0.000072 | 23.6 | possibly damaging | tolerated | Phenotype: Anterior segment dysgenesis 7, with sclerocornea (OMIM 269400) |
|  | PXDN | C Het | | Chr2:  g.1652962G>C | NM_012293.3:  c.2590C>G | NM_012293.3:  p.Pro864Ala | 0.000094 | 17.04 | benign | tolerated |  |
|  | SDK1 | C Het | | Chr7:  g.4213965A>G | NM_152744.4:  c.4912A>G | NM_152744.4:  p.Ile1638Val | 0.000428 | 8.781 | benign | tolerated | Adhesion molecule that promotes lamina-specific synaptic connections in the retina. Minimal brain expression. One homozygote for Ile1638Val in gnomAD. |
|  | SDK1 | C Het | | Chr7:  g.4260967C>T | NM_152744.4:  c.5798C>T | NM_152744.4:  p.Thr1933Met | 0.000226 | 24.2 | probably damaging | tolerated |  |
|  | TKTL2 | *De novo* | | Chr4:  g.164394223C>T | NM_032136.5:  c.664G>A | NM_032136.5:  p.Val222Met | 0 | 22.7 | probably damaging | deleterious | Expressed in testes. Role in cancer cell proliferation. Mice homozygous for null allele exhibit normal fecundity. |

Underlined, not OMIM morbid; C Het, Compound heterozygous; Hom, Homozygous; N/A, not applicable; CNV, copy number variant; AF, allele frequency; KO, knock out; LOF, loss of function. Shading indicates compound heterozygous variant pairs.

Ensembl transcripts used if no equivalent RefSeq available.

Data gathered from OMIM (https://www.omim.org/), Uniprot (https://www.uniprot.org/), MGI database (http://www.informatics.jax.org/), GnomAD https://gnomad.broadinstitute.org/), polyphen (http://genetics.bwh.harvard.edu/pph2/), SIFT/provean (http://provean.jcvi.org/index.php), CADD (https://cadd.gs.washington.edu/), GTEX (https://www.gtexportal.org/home/), Unique (https://rarechromo.org/), decipher (<https://www.deciphergenomics.org/>), Pubmed (https://pubmed.ncbi.nlm.nih.gov/).

# **Supplementary table 4: Gastrointestinal phenotypes in animal models with disruption of phosphatidylinositol metabolism**

Table showing published associations between gastrointestinal pathology in animal models and disruption of phosphatidylinositol metabolism.

Bold text indicates symptoms or signs that overlap with features seen in human patients with MIA and/or inflammatory bowel disease and immunodeficiency due to biallelic pathogenic variants in *TTC7A* or *PI4KA*.

| **GENE** | **ANIMAL MODEL** | **BOWEL- MACROSCOPIC** | **BOWEL-**  **MICROSCOPIC** | **ADDITIONAL INFO** | **OUTCOME** |
| --- | --- | --- | --- | --- | --- |
| *CDIPT ^18^* | Zebrafish | **Intestines small with diffuse inflammation** from day 6 post fertilisation | **Disorganised epithelial proliferation, abnormal villous architecture, leukocyte aggregation and an intestinal lumen filled with basophilic plaques** | **Hepatomegaly** **and steatosis** | Fish died shortly after development of bowel symptoms |
| *PIK3C3 ^19^* | Zebrafish | **Inflammatory bowel** disease- like | Failure of maintenance of **intestinal epithelium** from day 7 post fertilisation |  |  |
| *PI3K ^20^* | Mice | **Shorter, thicker colons** with chronic **colitis** |  | **Defective B and T cell signaling** |  |
| *PI4KA* | Mice^21,22^ | **Distended** gastrointestinal tract^21^ | Widespread gastrointestinal **epithelial cell** **degeneration/ necrosis^21, 22^** with evidence of regeneration in stomach, and small and large intestine^22^ |  | Conditional knock out mice died or were moribund and humanly euthanized by day 8^21,22^ |

# **References**

1. Fasham J, Leslie JS, Harrison JW, et al. No association between SCN9A and monogenic human epilepsy disorders. *PLoS Genet*. Nov 2020;16(11):e1009161. doi:10.1371/journal.pgen.1009161

2. Helman G, Lajoie BR, Crawford J, et al. Genome sequencing in persistently unsolved white matter disorders. *Ann Clin Transl Neurol*. Jan 2020;7(1):144-152. doi:10.1002/acn3.50957

3. Pagnamenta AT, Howard MF, Wisniewski E, et al. Germline recessive mutations in PI4KA are associated with perisylvian polymicrogyria, cerebellar hypoplasia and arthrogryposis. *Hum Mol Genet*. Jul 1 2015;24(13):3732-41. doi:10.1093/hmg/ddv117

4. Ferla MP, Pagnamenta AT, Damerell D, Taylor JC, Marsden BD. MichelaNglo: sculpting protein views on web pages without coding. *Bioinformatics*. May 1 2020;36(10):3268-3270. doi:10.1093/bioinformatics/btaa104

5. Rose AS, Bradley AR, Valasatava Y, Duarte JM, Prlic A, Rose PW. NGL viewer: web-based molecular graphics for large complexes. *Bioinformatics*. Nov 1 2018;34(21):3755-3758. doi:10.1093/bioinformatics/bty419

6. Berman H, Henrick K, Nakamura H. Announcing the worldwide Protein Data Bank. *Nat Struct Biol*. Dec 2003;10(12):980. doi:10.1038/nsb1203-980

7. Berman HM, Westbrook J, Feng Z, et al. The Protein Data Bank. *Nucleic acids research*. Jan 1 2000;28(1):235-42. doi:10.1093/nar/28.1.235

8. Lees JA, Zhang Y, Oh MS, et al. Architecture of the human PI4KIIIalpha lipid kinase complex. *Proc Natl Acad Sci U S A*. Dec 26 2017;114(52):13720-13725. doi:10.1073/pnas.1718471115

9. UniProt C. UniProt: a worldwide hub of protein knowledge. *Nucleic acids research*. Jan 8 2019;47(D1):D506-D515. doi:10.1093/nar/gky1049

10. Landau M, Mayrose I, Rosenberg Y, et al. ConSurf 2005: the projection of evolutionary conservation scores of residues on protein structures. *Nucleic acids research*. Jul 1 2005;33(Web Server issue):W299-302. doi:10.1093/nar/gki370

11. Ashkenazy H, Abadi S, Martz E, et al. ConSurf 2016: an improved methodology to estimate and visualize evolutionary conservation in macromolecules. *Nucleic acids research*. Jul 8 2016;44(W1):W344-50. doi:10.1093/nar/gkw408

12. Kelley LA, Mezulis S, Yates CM, Wass MN, Sternberg MJ. The Phyre2 web portal for protein modeling, prediction and analysis. *Nat Protoc*. Jun 2015;10(6):845-58. doi:10.1038/nprot.2015.053

13. Waterhouse A, Bertoni M, Bienert S, et al. SWISS-MODEL: homology modelling of protein structures and complexes. *Nucleic acids research*. Jul 2 2018;46(W1):W296-W303. doi:10.1093/nar/gky427

14. Chaudhury S, Lyskov S, Gray JJ. PyRosetta: a script-based interface for implementing molecular modeling algorithms using Rosetta. *Bioinformatics*. Mar 1 2010;26(5):689-91. doi:10.1093/bioinformatics/btq007

15. Hladilkova E, Barøy T, Fannemel M, et al. A recurrent deletion on chromosome 2q13 is associated with developmental delay and mild facial dysmorphisms. *Molecular cytogenetics*. 2015;8:57. doi:10.1186/s13039-015-0157-0

16. Yu HE, Hawash K, Picker J, et al. A recurrent 1.71 Mb genomic imbalance at 2q13 increases the risk of developmental delay and dysmorphism. *Clin Genet*. Mar 2012;81(3):257-64. doi:10.1111/j.1399-0004.2011.01637.x

17. Riley KN, Catalano LM, Bernat JA, et al. Recurrent deletions and duplications of chromosome 2q11.2 and 2q13 are associated with variable outcomes. *Am J Med Genet A*. Nov 2015;167a(11):2664-73. doi:10.1002/ajmg.a.37269

18. Thakur PC, Davison JM, Stuckenholz C, Lu L, Bahary N. Dysregulated phosphatidylinositol signaling promotes endoplasmic-reticulum-stress-mediated intestinal mucosal injury and inflammation in zebrafish. *Disease models & mechanisms*. Jan 2014;7(1):93-106. doi:10.1242/dmm.012864

19. Zhao S, Xia J, Wu X, et al. Deficiency in class III PI3-kinase confers postnatal lethality with IBD-like features in zebrafish. *Nat Commun*. Jul 6 2018;9(1):2639. doi:10.1038/s41467-018-05105-8

20. Uno JK, Rao KN, Matsuoka K, et al. Altered macrophage function contributes to colitis in mice defective in the phosphoinositide-3 kinase subunit p110delta. *Gastroenterology*. Nov 2010;139(5):1642-53, 1653.e1-6. doi:10.1053/j.gastro.2010.07.008

21. Vaillancourt FH, Brault M, Pilote L, et al. Evaluation of phosphatidylinositol-4-kinase IIIalpha as a hepatitis C virus drug target. *J Virol*. Nov 2012;86(21):11595-607. doi:10.1128/JVI.01320-12

22. Bojjireddy N, Botyanszki J, Hammond G, et al. Pharmacological and genetic targeting of the PI4KA enzyme reveals its important role in maintaining plasma membrane phosphatidylinositol 4-phosphate and phosphatidylinositol 4,5-bisphosphate levels. *J Biol Chem*. Feb 28 2014;289(9):6120-32. doi:10.1074/jbc.M113.531426
